# Two functionally redundant sources of retinoic acid secure spermatogonia differentiation in the seminiferous epithelium

**DOI:** 10.1242/dev.170225

**Published:** 2019-01-04

**Authors:** Marius Teletin, Nadège Vernet, Jianshi Yu, Muriel Klopfenstein, Jace W. Jones, Betty Féret, Maureen A. Kane, Norbert B. Ghyselinck, Manuel Mark

**Affiliations:** 1 Institut de Génétique et de Biologie Moléculaire et Cellulaire (IGBMC), Département de Génétique Fonctionnelle et Cancer, Centre National de la Recherche Scientifique (CNRS UMR7104), Institut National de la Santé et de la Recherche Médicale (INSERM U1258), Université de Strasbourg (UNISTRA), 1 rue Laurent Fries, F-67404 Illkirch Cedex, France; 2 Service de Biologie de la Reproduction, Hôpitaux Universitaires de Strasbourg (HUS), France; 3 Department of Pharmaceutical Sciences, University of Maryland School of Pharmacy, Baltimore, MD 21201, USA

**Keywords:** Spermatogenesis, Puberty, Immunohistochemistry, *Lgals1*, *Aldh8a1* knockout

## Abstract

In mammals, all-*trans* retinoic acid (ATRA) is instrumental to spermatogenesis. It is synthesized by two retinaldehyde dehydrogenases (RALDH) present in both Sertoli cells (SCs) and germ cells (GCs). In order to determine the relative contributions of each source of ATRA, we have generated mice lacking all RALDH activities in the seminiferous epithelium (SE). We show that both the SC- and GC-derived sources of ATRA cooperate to initiate and propagate spermatogenetic waves at puberty. In adults, they exert redundant functions and, against all expectations, the GC-derived source does not perform any specific roles despite contributing to two-thirds of the total amount of ATRA present in the testis. The production from SCs is sufficient to maintain the periodic expression of genes in SCs, as well and the cycle and wave of the SE, which account for the steady production of spermatozoa. The production from SCs is also specifically required for spermiation. Importantly, our study shows that spermatogonia differentiation depends upon the ATRA synthesized by RALDH inside the SE, whereas initiation of meiosis and expression of STRA8 by spermatocytes can occur without ATRA.

## Abstract (precis)

**Summary:** All-trans retinoic acid made by Sertoli cells is instrumental to spermatogenesis and is specifically required for spermatid release.

## INTRODUCTION

Mammalian spermatogenesis is the tightly regulated germ cell (GC) differentiation process that takes place in the seminiferous epithelium (SE) of the testis under the control of somatic, Sertoli, cells (SCs) (reviewed by de Rooij and Russell, 2000; Griswold, 2016). In the mouse, stem spermatogonia are among the A undifferentiated (A_undiff_) spermatogonia, endowed with self-renewal capacities. They differentiate into A1 spermatogonia, committed towards meiosis, which divide to generate successively A2, A3 and A4 spermatogonia, referred to as A differentiating (A_diff_) spermatogonia, then intermediate (Int) and B spermatogonia. The latter divide to generate preleptotene spermatocytes that initiate meiosis and subsequently go through the stages of primary (leptotene, zygotene, pachytene and diplotene) and secondary spermatocytes. Round spermatids, the end-products of meiosis, are subject to morphological changes and give rise to spermatozoa. The transition from A_undiff_ to A_diff_ spermatogonia proceeds in a periodic manner, every 8.6 days, whereas their descendants are generated and displaced at a constant pace towards the lumen of the seminiferous tubules. These processes result in the formation, at a given point along a seminiferous tubule, of stratified, recurring, GC associations of fixed composition, known as the 12 (I to XII) stages of the SE cycle (de Rooij, 1998; Russell, 1990). This cycle, as well as the repetitive patterning of the epithelial stages along the length of a seminiferous tubule, called the wave of the SE, represent the keys for asynchronous GC differentiation, allowing the constant production of spermatozoa by the testis. Precise associations between spermatogonia and spermatocytes are established during pubertal spermatogenesis, which initiates around postnatal day 2 (P2), and is completed by P35 (Drumond et al., 2011; Kluin et al., 1982; Yoshida et al., 2006).

A wealth of data indicate that all-trans retinoic acid (ATRA), the active metabolite of vitamin A (vitA) is instrumental to spermatogenesis. First, vitA-deficiency studies, as well as analyses of testes in which ATRA synthesis is inhibited either genetically or chemically, all demonstrate that ATRA is a key regulator of the A_undiff_ to A_diff_ transition (Ghyselinck et al., 2006; Hogarth et al., 2013; Morales and Griswold, 1987; Raverdeau et al., 2012; Schrans-Stassen et al., 1999; Tong et al., 2013; van Pelt and de Rooij, 1991). ATRA has also been involved in controlling the entry of male germ cells into meiosis (MacLean et al., 2007; Raverdeau et al., 2012; reviewed by Bowles and Koopman, 2007; Griswold, 2016; Teletin et al., 2017), and is a major player in spermiation, the process whereby mature spermatids are translocated to the luminal edge of the SE, then released (Ghyselinck et al., 2006; Huang and Marshall, 1983; Raverdeau et al., 2012). Last, several observations support the notion that ATRA regulates the SE cycle and wave: decreased retinoid concentrations disrupt the SE cycle, as assessed by the occurrence of abnormal epithelial stages that lack one or several generations of GC in vitA-deficient mice (Ghyselinck et al., 2006), whereas exposure of vitA-deficient rats and of neonatal mice to exogenously administered ATRA eliminates the wave of the SE, resulting in a ‘synchronized’ spermatogenesis (Davis et al., 2013; Morales and Griswold, 1987).

Under physiological conditions, ATRA available to GCs is derived from local synthesis, because of the presence of a catabolic barrier separating the SE from the interstitial tissue (Kurlandsky et al., 1995; Teletin et al., 2017; Vernet et al., 2006b). Inside the SE, ATRA is synthesized from vitA through a two-step oxidation that includes the activities of retinaldehyde dehydrogenases (RALDH1 to RALDH3, encoded by *Aldh1a1* to *Aldh1a3* genes). Immunohistochemistry (IHC) and *in situ* hybridization (ISH) analyses have shown that RALDH1 and RALDH2 are the main ATRA-synthesizing enzymes in SCs and GCs, respectively, where their expression patterns in the adult testis are both periodic, non-overlapping and highest at specific stages of the SE cycle (Sugimoto et al., 2012; Teletin et al., 2017; Vernet et al., 2006b). SCs robustly express RALDH1 from the fetal period onwards (Bowles et al., 2009; Kent et al., 2016; Teletin et al., 2017) and they also contain low levels of *Aldh1a2* transcripts after birth (Vernet et al., 2006b). During pubertal testis development, spermatocytes are the first GCs to express *Aldh1a2* (Kent et al., 2016; Raverdeau et al., 2012; Vernet et al., 2006b). ATRA produced by RALDH then binds to and activates its nuclear receptors (reviewed by Mark et al., 2015).

In a previous study, we have established, through the phenotypic analysis of mutant mice lacking all RALDH activities in SCs (*Aldh1a1-3^Ser−/−^* mutants), that ATRA produced by SCs initiates the transition from A_undiff_ to A_diff_ spermatogonia and thus the pubertal spermatogenetic wave. Spermatogenesis can be stably rescued by a single injection of ATRA, pointing to potentially important roles of ATRA synthesized by GCs, at least when the SC-derived source is impaired (Raverdeau et al., 2012). Recent experiments in which either exogenous ATRA was administered to adult mice or their testes were depleted from meiotic cells conclude that ATRA from pachytene spermatocytes is required for the initiation of spermatid elongation and for the release of spermatozoa (Endo et al., 2017). However, chemical approaches to change ATRA levels in a tissue do not necessarily reflect endogenous requirements or regulation of ATRA, and genetic approaches are considered as highly desirable to validate what is reported in treated animals (Cunningham and Duester, 2015; Duester, 2017). Thus, to gain further insights into the contribution of the GC-derived source of ATRA to spermatogenesis and spermiation, we have generated and analyzed mouse mutants lacking all RALDH activities in GCs (i.e. *Aldh1a1-3^Germ−/−^* mutants) or in both GCs and SCs (i.e. *Aldh1a1-3^Germ−/−;Ser−/−^* compound mutants). Comparison of the mutant phenotypes indicates that ATRA from SCs and GCs exerts both specific and redundant functions in male reproduction. It notably shows that: (1) spermatogonia differentiation crucially depends upon ATRA synthesized within the SE; (2) neither initiation of meiosis nor expression of STRA8 in spermatocytes depends upon ATRA in the SE; and (3) the GC-derived source of ATRA is fully dispensable for spermiation, whereas the SC-derived source is indispensable.

## RESULTS

### *Aldh1a* gene ablation in germ cells decreases testicular ATRA concentration

**Fig. 1.**
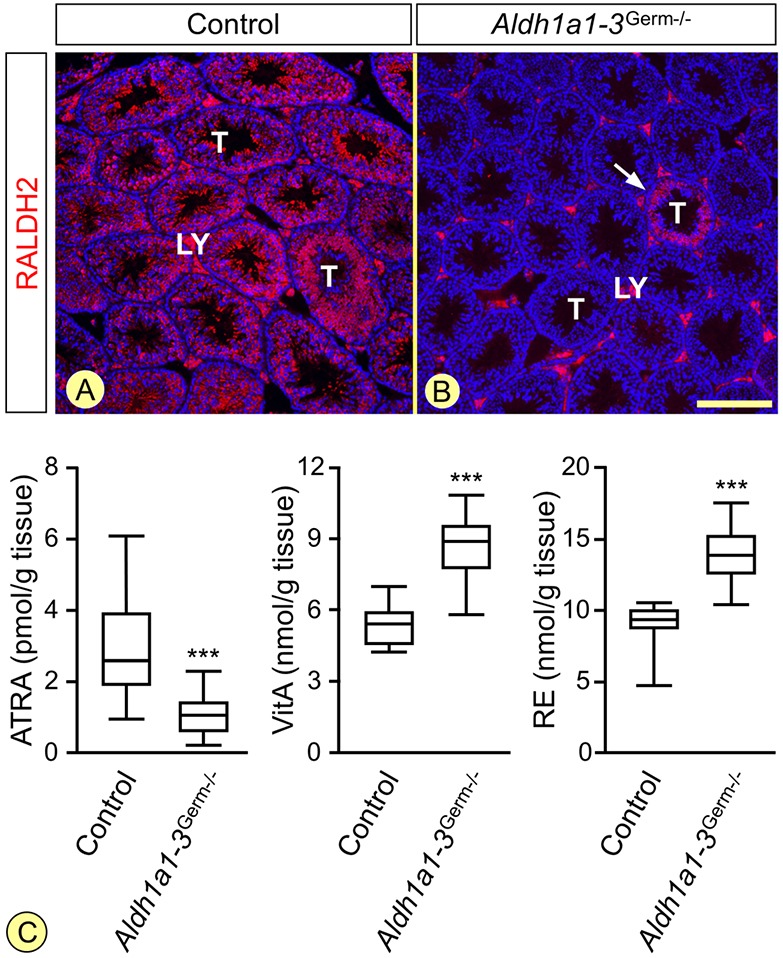
**The *Aldh1a1-3^Germ−/−^* mutation suppresses RALDH2 expression in germ cells and alters testicular retinoid metabolism.** (A,B) Immunohistochemical detection of RALDH2 (red signal) on histological sections from 8-week-old control and *Aldh1a1-3^Germ−/−^* mice. The white arrow in B indicates the only seminiferous tubule (T) cross-section, out of the 230, that still expresses RALDH2. The signal observed in Leydig cells (LY) is unspecific, as RALDH2 transcripts are not detected in these cells (Vernet et al., 2006b). Nuclei were counterstained with DAPI (blue signal). Scale bar: 160 µm. (C) Box-and-whisker plots showing the testicular concentrations of ATRA, vitA and retinyl esters (RE) in 9- to 10-week-old *Aldh1a1-3^Germ−/−^* (*n*=16) and control (*n*=14) mice. The boxes indicate the upper and lower quartiles, the lines inside mark the medians, and the whiskers delineate the highest and lowest values. ***P<0.001, Student's *t*-test.

We first confirmed ISH data (Vernet et al., 2006b) indicating that RALDH2 is the main, if not the sole, ALDH1A isotype expressed in GCs of the wild-type testis (Figs S1 and S2). We nevertheless generated a mouse model with a GC-specific ablation of all three isotypes, in order to prevent any possible functional compensation by another RALDH expressed at a low level. Ablation of RALDH was assessed in 8- to 10-week-old *Aldh1a1-3^Germ−/−^* mutants (*n*=4) by IHC. Only 0.5 to 2% of the seminiferous tubule cross-sections present on a transverse histological section of the testis reacted with the anti-RALDH2 antibody, as opposed to 100% in control testes (Fig. 1A,B). Furthermore, out of 94 pups born from four *Aldh1a1-3^Germ−/−^* adult males mated with wild-type females, 93 (i.e. 98.9%) exhibited one excised allele of *Aldh1a1*, *Aldh1a2* and *Aldh1a3* genes, indicating efficient ablation of RALDH-coding genes in *Aldh1a1-3^Germ−/−^* males.

The average concentration of testicular ATRA was significantly lower in 9- to 10-week-old *Aldh1a1-3^Germ−/−^* mutants (1.14±0.64 pmol/gram of tissue) than in controls (3.09±1.63 pmol/gram of tissue; Fig. 1C). Interestingly, inhibition of ATRA synthesis influenced the metabolism of upstream retinoids, as both retinol and retinyl ester levels were higher in mutant than in control testes (Fig. 1C). Altogether, these results indicate that RALDH are efficiently excised in *Aldh1a1-3^Germ−/−^* testes, yielding a 63% decrease of ATRA concentration.

### RALDH activity in germ cells is dispensable for the seminiferous epithelium cell cycle and wave, as well as for the periodic release of mature spermatids

The weights of *Aldh1a1-3^Germ−/−^* adult testes (98.7±10.4 mg) did not differ significantly (*P*=0.15) from those of their control littermates (103.2±10.8 mg). This was also the case for the diameters of seminiferous tubules (179±10 µm in *Aldh1a1-3^Germ−/−^* testes versus 182±5 µm in control testes, *P*=0.62). Along the same lines, the mean number of preleptotene (prL) and leptotene (L) spermatocytes (at stages VII-VIII) or early zygotene (Z) spermatocytes (at stage X) per mm of tubule circumference were similar in control and *Aldh1a1-3^Germ−/−^* mutants (prL/L, 67±3 versus 67±5, *P*=0.98; Z, 66±3 versus 68±3, *P*=0.66). The same applied for Sertoli cells (35±4 versus 34±6, *P*=0.88).

**Fig. 2.**
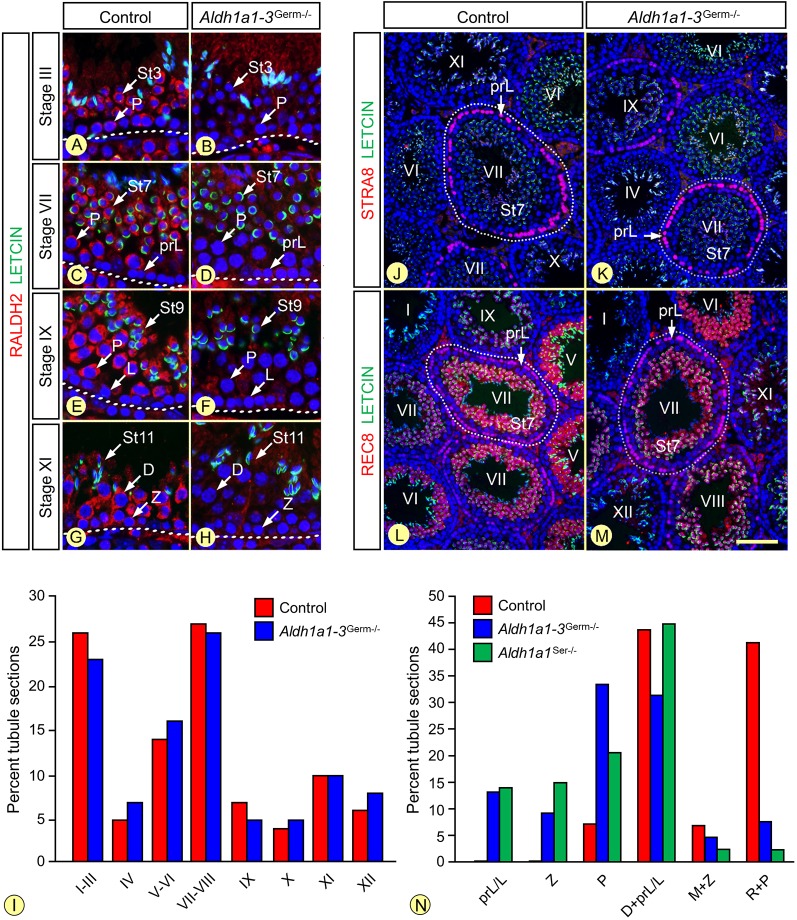
**The adult *Aldh1a1-3^Germ−/−^* mutant SE is normal.** (A-H) Detection of RALDH2 (red signal) on histological sections from 8-week-old control and *Aldh1a1-3^Germ−/−^* mice. RALDH2 is completely absent in the mutant GC. (I) Relative frequencies of the stages of the SE in 9- to 10-week-old control (red bars, *n*=10) and *Aldh1a1-3^Germ−/−^* (blue bars, *n*=4) testes. (J-M) Detection of STRA8 and REC8 (red signals) on histological sections from 8-week-old control and *Aldh1a1-3^Germ−/−^* mice. In both cases, STRA8 is expressed in preleptotene and leptotene spermatocytes at epithelial stages VII to IX. REC8 is detected in preleptotene spermatocytes at stages VII and VIII, and in spermatids at stages V to IX. Nuclei were counterstained by DAPI (blue signal) and the acrosomal system by Alexa Fluor 488-conjugated peanut agglutinin (green signal), allowing proper staging. Roman numerals designate stages of the SE cycle. The dotted lines indicate the periphery of seminiferous tubules. (N) Ablation of either the SC- or the GC-derived source of ATRA delays spermatogenesis. Mean percentages of seminiferous tubule sections in P21 control (red bars), *Aldh1a1-3^Germ−/−^* (blue bars) and *Aldh1a1^Ser−/−^* (green bars) testes containing: preleptotene or leptotene spermatocytes and a peripheral row of spermatogonia (prL/L); zygotene spermatocytes and a peripheral row of spermatogonia (Z); early pachytene spermatocytes and a peripheral row of spermatogonia (P); late pachytene or diplotene spermatocytes with a peripheral row of either preleptotene or leptotene spermatocytes (D+prL/L); meiotic metaphases with a peripheral row of zygotene spermatocytes (M+Z); round spermatids with a peripheral row of pachytene spermatocytes (R+P). D, prL, L, P and Z indicate diplotene, preleptotene, leptotene, pachytene and zygotene spermatocytes, respectively; St3 to St11, steps of spermatid maturation. Scale bar: 30 µm in A-H; 80 µm in J-M.

Analyses by ISH and IHC suggest that RALDH2 activity in GCs varies periodically during the SE cycle (Sugimoto et al., 2012; Vernet et al., 2006b), thereby generating periodic variations of ATRA concentrations (Hogarth et al., 2015; Endo et al., 2017), the impairment of which is currently held responsible for alterations of the SE cycle and wave (Endo et al., 2017; Hasegawa and Saga, 2012; Hogarth et al., 2015; Snyder et al., 2011; Sugimoto et al., 2012). In view of these data, we carefully analyzed the *Aldh1a1-3^Germ−/−^* SE according to histological criteria (see Materials and Methods). The full representation of all the GC populations characteristic of the 12 epithelial stages of the SE were present in *Aldh1a1-3^Germ−/−^* mutants analyzed at 8 to 10 weeks or 12-18 months of age (Fig. 2A-H). We further reasoned that loss of RALDH activities in GCs could synchronize the SE in *Aldh1a1-3^Germ−/−^* testes. Against this view, the proportions of the 12 epithelial stages were similar in *Aldh1a1-3^Germ−/−^* and control testes (Fig. 2I). Accordingly, their mean synchronization factors (van Beek and Meistrich, 1990) were highly similar (1.07±0.01 and 1.08±0.01, in controls and *Aldh1a1-3^Germ−/−^* mutants, respectively; *P*=0.51). In addition, the periodic expression patterns of *Stra8* and *Rec8*, two independent meiotic markers of the GCs response to ATRA (Hogarth et al., 2013; Koubova et al., 2014) were remarkably similar in *Aldh1a1-3^Germ−/−^* and control testes (Fig. 2J-M; Fig. S4). Thus, RALDH activity in GCs appears dispensable to the control of the SE cycle and wave.

SCs display periodic changes in gene expression, which are associated with specific stages of the SE cycle, a phenomenon referred to as the SC cycle. This periodic activity of SCs was investigated through analysis of GATA1, androgen receptor (AR), STRA6 and claudin 3 (CLDN3) proteins, as well as *Lgals1* transcripts (Vernet et al., 2006a). All of them displayed similar, periodic, expression patterns in *Aldh1a1-3^Germ−/−^* and control testes (Figs S4 and S5), notably *Stra6* and *Lgals1*, both of which are ATRA responsive (Bouillet et al., 1997; Lu et al., 2000). Thus, RALDH activity in GCs appears dispensable to the control of the SC cycle.

Spermiation is very sensitive to retinoid deficiency (Ghyselinck et al., 2006; Huang and Marshall, 1983; Raverdeau et al., 2012). In this context, it is noteworthy that *Aldh1a1-3^Germ−/−^* mutants displayed a fully normal spermiation, as indicated by the alignment of all mature spermatid heads at the luminal edge of the SE at stages VII and VIII, and by the absence of retained spermatids at stage IX (Fig. 2C-F).

We next assessed global mRNA expression changes to identify any subtle defects in adult *Aldh1a1-3^Germ−/−^* mutant testes. Surprisingly, deletion of *Aldh1*a genes in GCs caused deregulation of a limited number of genes: only 43 genes, including *Aldh1a2*, showed significant (*P*<0.05) expression changes of 1.5-fold or greater (Table 1). The levels of mRNA for *Aldh1a1*, *Aldh1a3* and all other enzymes involved in retinoid metabolism were unchanged. The expression changes were verified by quantitative, real-time, RT-PCR for six selected genes (Fig. S6). Gene ontology analysis did not identify alteration of any pathways, notably those important for SC function and GC differentiation, with the notable exceptions of *Stra8* and *Lgals1*.

**Table 1.**
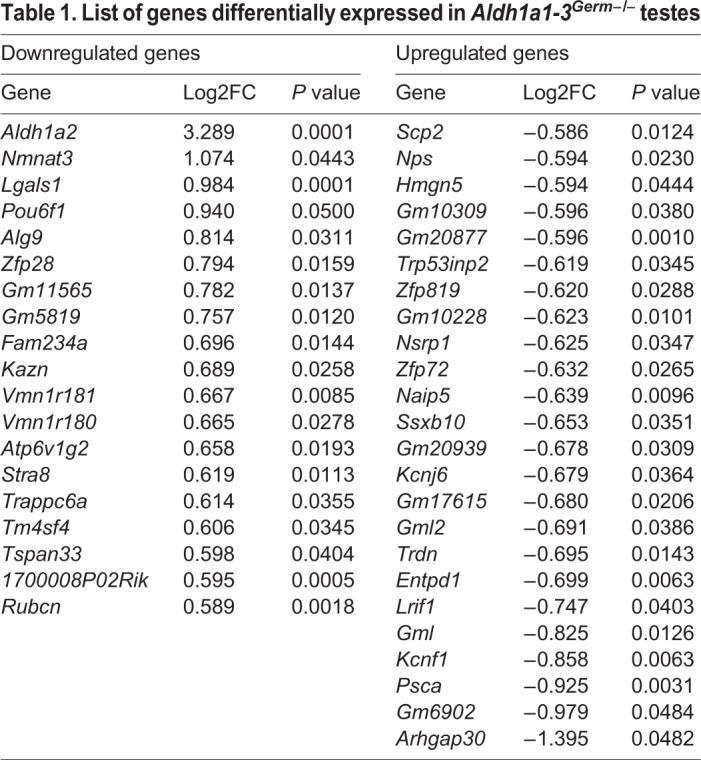
**List of genes differentially expressed in *Aldh1a1-3^Germ−/−^* testes**

Altogether, our data indicate that, unexpectedly, the loss of all RALDH isotypes in GCs does not affect homeostatic spermatogenesis or spermiation throughout the male reproductive lifespan. Accordingly, *Aldh1a1-3^Germ−/−^* males were normally fertile. Interestingly, a very recent work also reports a normal testis histology and a normal fertility in male mice lacking solely RALDH2 in germ cells (Beedle et al., 2018).

### Both the Sertoli and germ cell-derived sources of ATRA promote the progression of pubertal spermatogenesis

We next analyzed the SE of *Aldh1a1-3^Germ−/−^* mutants at P21, which is 1 day after the emergence of post-meiotic cells (i.e. round spermatids; Drumond et al., 2011). To classify the cross-sections of seminiferous tubules, we took into account both the most advanced GC types, which occupy central positions within the tubules, and the next generation of GCs present at the periphery. We reasoned that the progression of spermatogenesis was retarded in cross-sections harboring a single generation of spermatocytes, when compared with those containing either two generations of spermatocytes or round spermatids. We found that *Aldh1a1-3^Germ−/−^* mutants displayed almost half as many (*P*<0.01) tubule sections containing two generations of meiotic and/or post-meiotic cells as their control littermates (Fig. 2N, compare blue with red bars). These data indicate that progression of spermatogenesis during puberty is delayed by several days in *Aldh1a1-3^Germ−/−^* mutants.

We then examined the possibility that the SC-derived source of ATRA could also participate in the progression of the pubertal spermatogenesis. To do so, the *Aldh1a1-3^Ser−/−^* mutants were unsuitable because their GCs cannot progress beyond the A_undiff_ spermatogonia stage unless they are exposed to exogenous ATRA (Raverdeau et al., 2012). We therefore focused on *Aldh1a1^Ser−/−^* males, in which only RALDH1 is absent from SCs. Similar to *Aldh1a1^−/−^* mutants (Matt et al., 2005), *Aldh1a1^Ser−/−^* males were normally fertile. Their testes displayed a delay in spermatogenesis similar to that observed in *Aldh1a1-3^Germ−/−^* mutants, as only half of their tubule sections contained two generations of meiotic and/or post-meiotic cells at P21 (*P*<0.01, Fig. 2N, compare green with red bars). Altogether, these results indicate that progression of pubertal spermatogenesis is supported by both the GC- and SC-derived sources of ATRA.

### Synchronized RALDH-deficient testes as a model system to study spermatogonia differentiation

Several lines of evidence indicate that ATRA signaling does not impact the duration of meiosis (Endo et al., 2017; Gely-Pernot et al., 2012; Ghyselinck et al., 2006). We thus assumed that the delayed spermatogenesis observed in RALDH-deficient prepubertal testes could be caused by a delayed onset and/or by the lengthening of the spermatogonia differentiation process. To test for this hypothesis, we compared the timeline of spermatogonia differentiation in *Aldh1a1-3^Germ−/−^*, *Aldh1a1-3^Ser−/−^* and control testes.

**Fig. 3.**
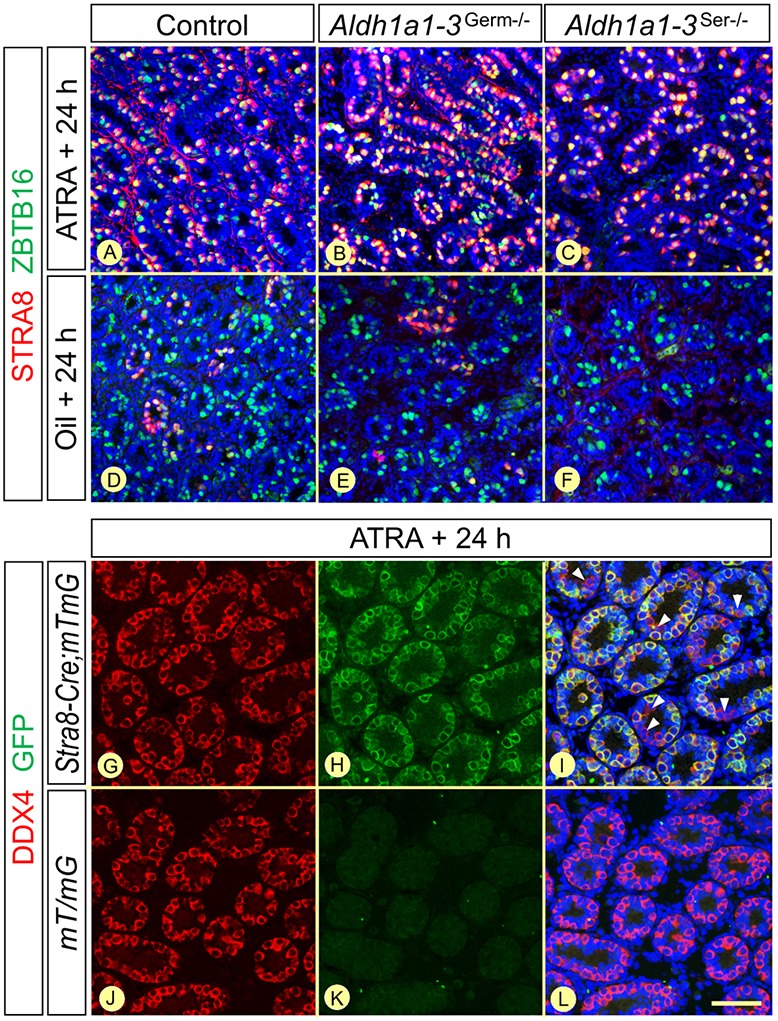
**Synchronization of differentiation and Cre-mediated excision in spermatogonia.** (A-F) Detection of STRA8 (red signal) and ZBTB16 (green nuclear signal) on histological sections from control, *Aldh1a1-3^Germ−/−^* and *Aldh1a1-3^Ser−/−^* testes 24 h after the administration of ATRA or sunflower oil at P3. (G-L) Detection of DDX4 (red cytoplasmic signal) and GFP (green membranous signal) on histological sections from *Stra8-Cre;mT/mG* and *mT/mG* mice 24 h after the administration of ATRA at P3. Efficient *mT/mG* excision by *Stra8-Cre* is assessed by GFP expression in almost all spermatogonia (G-I). In contrast, GFP is never detected in the absence of *Stra8-Cre* (J-L). The arrowheads in I indicate a few spermatogonia in which *mTmG* is not excised. Scale bar: 80 µm in A-F; 70 µm in G-L.

We classified spermatogonia into three subtypes according to marker expression (see Materials and Methods): A_undiff_ (ZBTB16+; STRA8−; KIT−), A_diff_ (ZBTB16+; STRA8+; KIT+) and Int-B (ZBTB16−; STRA8−; KIT+) (Mark et al., 2015; Teletin et al., 2017; Chan et al., 2017; Fig. S3). Spermatocytes were identified based on nuclear morphologies (Russell, 1990) and on the expression of STRA8 and gH2AX (Fig. S7). To enable monitoring of spermatogonia differentiation, we synchronized this process by administering a single dose of ATRA at P3 (Davis et al., 2013). We assessed the efficacy of the treatment by counting the percentages of cells expressing STRA8, a marker of the transition from A_undiff_ to A_diff_ spermatogonia, 24 h after the administration of ATRA or its vehicle (sunflower oil). In *Aldh1a1-3^Germ−/−^*, *Aldh1a1-3^Ser−/−^* and control testes, more than 90% of the spermatogonia (including their precursor cells, the gonocytes, which are present up to about P6) expressed STRA8 following ATRA treatment, versus about 20% in *Aldh1a1-3^Germ−/−^* and control testes and none in *Aldh1a1-3^Ser−/−^* testes following vehicle treatment (Fig. 3A-F). These data indicate that exposure to ATRA at P3 drives the bulk of the A_undiff_ population to enter the differentiation pathway. In parallel, the pattern of Cre-mediated gene excision by *Tg(Stra8-cre)^1Reb^* in spermatogonia was assessed using the *mT/mG* reporter transgene, which directs expression of a membrane-targeted green fluorescent protein (mG) in cells that have experienced Cre-mediated deletion (Muzumdar et al., 2007). Interestingly, mG was detected in more than 90% of the spermatogonia 24 h after ATRA treatment at P3 (Fig. 3G-L), indicating efficient excision by Cre in these cells. We assume therefore that most, if not all, spermatogonia induced to differentiate upon ATRA administration at P3 in *Aldh1a1-3^Germ−/−^* mice lose RALDH-coding genes with the same efficiency. However, this cannot be directly substantiated by IHC as spermatogonia do not express RALDH (Kent et al., 2016; see below).

We then analyzed the progression of synchronized seminiferous tubules over a period covering three consecutive rounds of spermatogonia differentiation. As this first round was triggered by exogenous ATRA (see above), we assumed that it was not necessarily representative of the physiological situation, and did not analyze it in detail. In contrast, as ATRA administered to mouse pups is completely cleared from the testes within 4 days following its injection (Hogarth et al., 2015), the second and third rounds relied exclusively on endogenous ATRA produced inside the seminiferous tubules. We paid particular attention to the A_undiff_ to A_diff_ transition, and to the entry into meiosis, which are known to depend on ATRA (reviewed by Bowles and Koopman, 2007; Griswold, 2016; Teletin et al., 2017). In control mice, the timing of the second round of spermatogonia differentiation and its coordination with the temporal sequence of spermatocyte progression through the meiotic prophase, analyzed on a day by day basis, was very similar to that reported in prepubertal testis from untreated wild-type mice (Table 2, Fig. S7; Drumond et al., 2011). This indicates that our model of synchronized spermatogenesis is physiologically relevant.

**Table 2.**
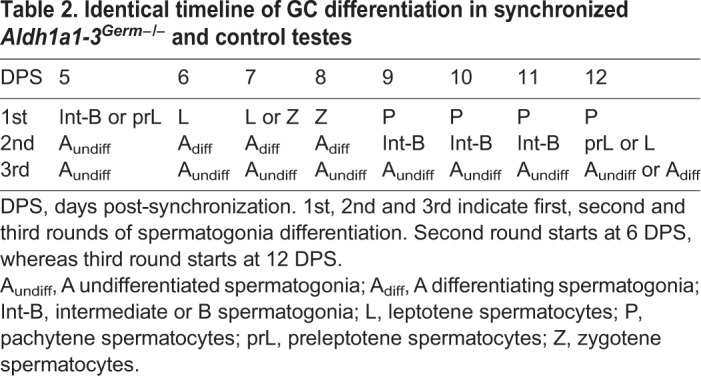
**Identical timeline of GC differentiation in synchronized *Aldh1a1-3^Germ−/−^* and control testes**

### Removing either the Sertoli or the germ cell-derived sources of ATRA does not alter the duration of spermatogonia differentiation

**Fig. 4.**
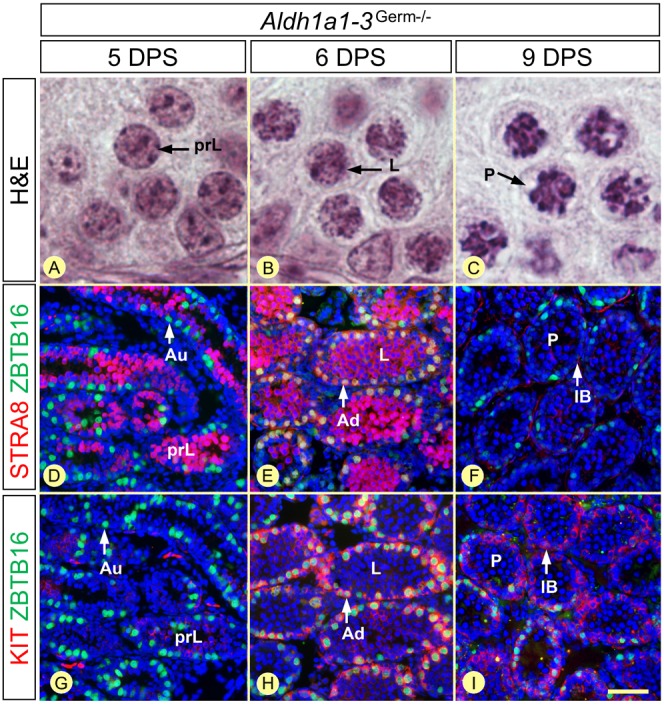
**Chronology of GC differentiation in *Aldh1a1-3^Germ−/−^* testes 5, 6 and 9 days after synchronization.** (A-C) Morphology of the spermatocytes, present at the center of the seminiferous tubules, assessed by Hematoxylin and Eosin staining. (D-I) Characterization of the spermatogonia populations, present at the periphery of the seminiferous tubules, by detection of ZBTB16 (green signal) and either STRA8 or KIT (red signals), as indicated. Nuclei were counterstained with DAPI (blue signal). D and G, and E and H are adjacent sections. Au, Ad and IB indicate A_undiff_, A_diff_ and intermediate or B spermatogonia, respectively; DPS, day post-synchronization; prL, L and P indicate preleptotene, leptotene and pachytene spermatocytes, respectively. Scale bar: 6 µm in A-C; 60 µm in D-I.

**Fig. 5.**
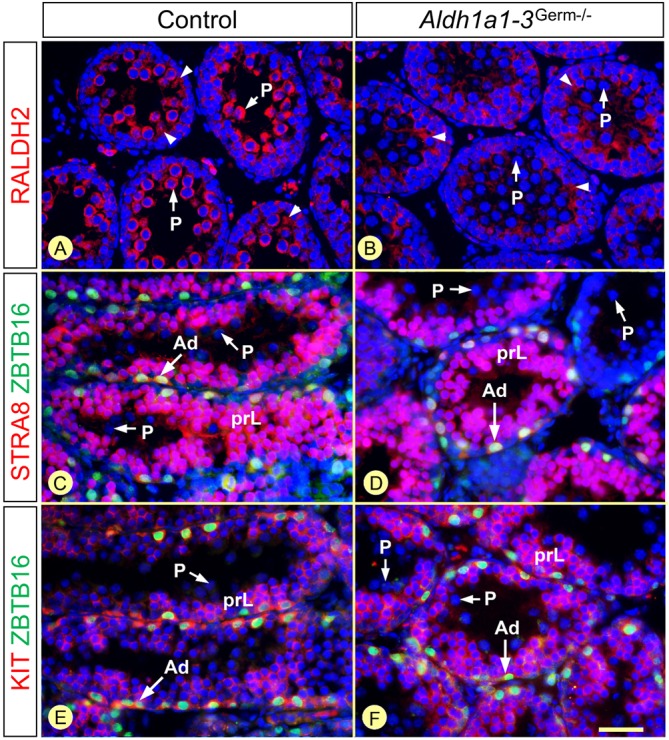
**The same types of GC are present in control and *Aldh1a1-3^Germ−/−^* testes 12 days after synchronization.** (A,B) Detection of RALDH2 (red signal). (C-F) Characterization of the spermatogonia and spermatocyte populations, present at the periphery of the seminiferous tubules, by detection of ZBTB16 (green signal) and either STRA8 or KIT (red signals), as indicated. Nuclei were counterstained with DAPI (blue signal). C and E are consecutive sections. Ad indicates A_diff_ spermatogonia; prL and P indicate preleptotene and pachytene spermatocytes, respectively. Arrowheads indicate RALDH2 in the cytoplasm of SCs. Scale bar: 30 µm.

In *Aldh1a1-3^Germ−/−^* mutants, spermatogonia, which were all at the A_undiff_ stage (ZBTB16+, STRA8−, KIT−) at 5 days post-synchronization (DPS), proceeded to A_diff_ (ZBTB16+, STRA8+, KIT+) and to Int-B (ZBTB16−, STRA8−, KIT+) stages at 6 and 9 DPS, respectively (Fig. 4). Spermatogonia differentiation ended at 12 DPS, when they transformed into preleptotene spermatocytes (Fig. 5; Table 2). During the same period, spermatocytes born from spermatogonia of the first, ATRA-induced, round progressed through meiosis at the center of the seminiferous tubules, where they appeared for the first time at 5 DPS, as STRA8+ preleptotene spermatocytes (Fig. 4). They gave rise to STRA8+ leptotene spermatocytes at 6 DPS, which differentiated into zygotene spermatocytes at 7 and 8 DPS, then into pachytene spermatocytes from 9 DPS onwards (Figs 4 and 5; Table 2). The timeline of the second round of spermatogonia differentiation and the tight correspondence between the chronology of appearance of the different spermatogonia subtypes and the maturation stages of spermatocytes was identical in control mice (Fig. 5; Fig. S7). In addition, the following A_undiff_-A_diff_ transition, indicative of the start of the third round of spermatogonia differentiation, occurred at 12 DPS both in *Aldh1a1-3^Germ−/−^* and control testes (Fig. 5; Table 2). We thus conclude that the developmental sequence of GC differentiation proceeds normally in the absence of ATRA synthesized by GCs.

In the adult SE, the time point at which spermatogonia undergo the A_undiff_-A_diff_ transition matches the appearance of preleptotene spermatocytes originating from the preceding generation of A_diff_ spermatogonia (de Rooij, 1998). Our results confirm previous morphological data indicating that the rigid, spatial and temporal, relationship between the commitment of spermatogonia to meiosis (A_undiff_ to A_diff_ transition) and their actual entry into meiosis (B spermatogonia to preleptotene spermatocyte transition) already exist in the prepubertal SE (Table 2; Drumond et al., 2011). They also demonstrate that this relationship is maintained in *Aldh1a1-3^Germ−/−^* testes at 12 DPS, despite the absence of the source of ATRA normally represented by pachytene spermatocytes (Fig. 5A,B).

**Fig. 6.**
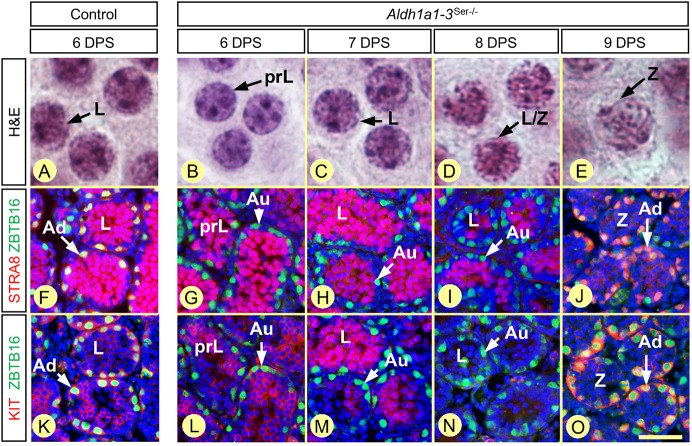
**Chronology of GC differentiation in *Aldh1a1-3^Ser−/−^* testes at 6, 7, 8 and 9 days after synchronization.** (A-E) Morphology of the spermatocytes, present at the center of the seminiferous tubules, assessed by Hematoxylin and Eosin staining in control (A) and mutant (B-E) testes. (F-O) Characterization of the spermatogonia populations, present at the periphery of the seminiferous tubules, assessed by detection of ZBTB16 (green signal) and either STRA8 or KIT (red signals), as indicated, in control (F,K) and mutant (G-J, L-O) testes. Nuclei were counterstained with DAPI (blue signal). Au and Ad indicate A_undiff_ and A_diff_ spermatogonia, respectively; DPS, day post-synchronization; prL, L and Z indicate preleptotene, leptotene and zygotene spermatocytes, respectively. Scale bar: 6 µm in A-E; 60 µm in F-O.

We have also monitored the timeline of spermatogonia differentiation in synchronized *Aldh1a1-3^Ser−/−^* mutants. The onset of the second round of spermatogonia differentiation, indicated by the A_undiff_ -A_diff_ transition, occurred at 9 DPS instead of 6 DPS in control testes (Fig. 6; Table 3). As a result of this delay, the first A_diff_ spermatogonia were associated with zygotene instead of leptotene spermatocytes (Fig. 6). Interestingly, spermatogonia rapidly lost the expression of STRA8 and thus spent only 1 day at the A_diff_ stage instead of 3 days in control testes (Table 3). Preleptotene spermatocytes derived from the second wave of spermatogonia differentiation appeared at 14 DPS, together with the A_undiff_-A_diff_ transition that is indicative of the start of the third round of spermatogonia differentiation (Table 3). Thus, although the timeline of *Stra8* expression is modified in spermatogonia of *Aldh1a1-3^Ser−/−^* mutants, the duration of their differentiation is similar to that observed in control and *Aldh1a1-3^Germ−/−^* testes (i.e. about 6 days). Altogether, these results demonstrate that the duration of spermatogonia differentiation is not influenced by low ATRA levels and are in agreement with previous studies indicating that STRA8 does not play an important role in spermatogonia differentiation (Endo et al., 2015).

**Table 3.**
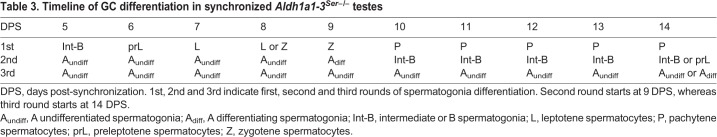
**Timeline of GC differentiation in synchronized *Aldh1a1-3^Ser−/−^* testes**

### The Sertoli and germ cell-derived sources of ATRA appear sequentially during normal puberty and recovery of spermatogenesis

**Fig. 7.**
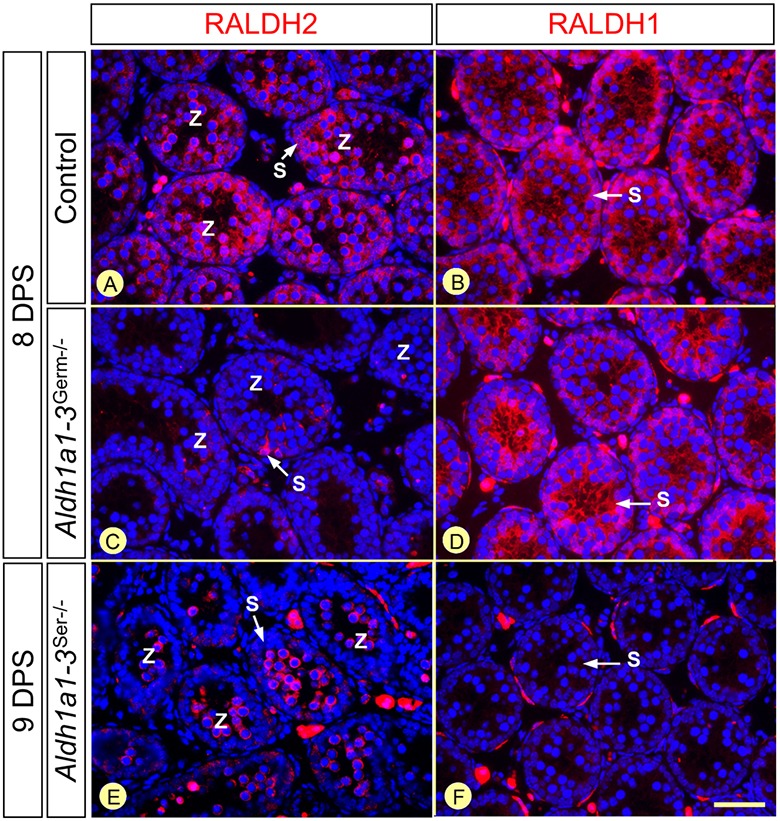
**Zygotene spermatocytes are the first GC type to express RALDH2 during testis development.** (A-F) Detection of RALDH1 and RALDH2 (red signals) at 8 or 9 DPS in *Aldh1a1-3^Germ−/−^*, *Aldh1a1-3^Ser−/−^* and control testes, as indicated. Nuclei were counterstained with DAPI (blue signal). Z, zygotene spermatocytes; S, Sertoli cell cytoplasm. Scale bar: 50 µm.

To identify precisely the earliest GC type expressing RALDH2 during testis development, we analyzed histological sections of *Aldh1a1-3^Germ−/−^*, *Aldh1a1-3^Ser−/−^* and control testes from 5 to 9 DPS. In controls, RALDH2 appeared for the first time in zygotene spermatocytes at 8 DPS (Fig. 7A). As expected, RALDH2-positive spermatocytes never occurred in *Aldh1a1-3^Germ−/−^* testes (Fig. 7C), whereas they appeared with a 1 day delay in synchronized *Aldh1a1-3^Ser−/−^* testes (Fig. 7E). These data indicate that, during normal testis development, zygotene spermatocytes, which appear at P9 during a normal wave of pubertal spermatogenesis (Kluin et al., 1982), represent the earliest GC-derived source of ATRA. This is much later than the Sertoli cell-derived source of ATRA, which is already present at P0 (Fig. S1). We further demonstrate that, contrary to previously published data (Kent et al., 2016), neither spermatogonia nor SCs express detectable amounts of RALDH4 protein (Figs S8 and S9).

### Low ATRA levels allow for the transition from spermatogonia to meiotic cells

**Fig. 8.**
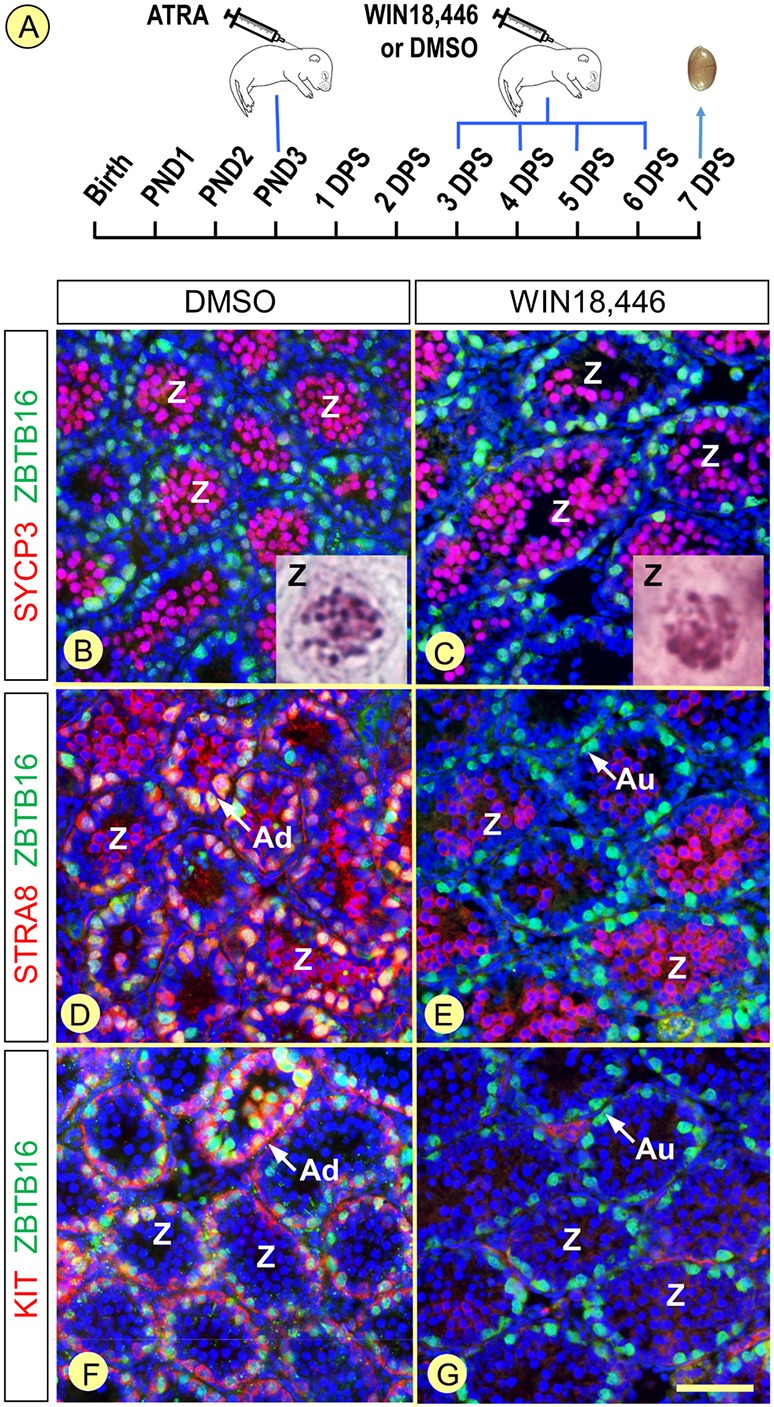
**Inhibition of spermatogonia differentiation, but not meiosis, upon exposure of synchronized *Aldh1a1-3^Germ−/−^* testes to WIN18,446.** (A) Pups were treated with ATRA at P3, with WIN18,446 or DMSO between 3 and 6 DPS, and their testes were analyzed at 7 DPS. (B,C) Spermatocytes expressing SYCP3 (red signal) and showing nuclear morphologies corresponding to zygotene spermatocytes (insets) fill the seminiferous tubules in both DMSO- and WIN18,446-treated pups. (D,E) STRA8-positive spermatogonia are present in DMSO-treated but totally absent in WIN18,446-treated pups (red signal at the periphery of the tubules). In contrast, STRA8 is expressed in zygotene spermatocytes in both DMSO- and WIN18,446-treated pups (red signal at the center of the tubules). (F,G) KIT-positive spermatogonia (red signal) are present in DMSO-treated but totally absent in WIN18,446-treated pups. Spermatogonia nuclei are stained using ZBTB16 (green signals), while all cell nuclei are stained using DAPI (blue signals). Au and Ad indicate A_undiff_ and A_diff_ spermatogonia, respectively. Z, zygotene spermatocytes. Scale bar: 60 µm.

Studies with agonists of the ATRA signaling pathway and measurement of ATRA concentrations have suggested that high ATRA levels in the SE coordinate the A_undiff_-A_diff_ transition and the timing of entry into meiosis (Endo et al., 2015; Hogarth et al., 2015). In synchronized *Aldh1a1-3^Ser−/−^* mutants, spermatogonia from the first round of differentiation entered meiosis with only a 1 day delay (i.e. it occurred at 6 DPS, instead of at 5 DPS in control and *Aldh1a1-3^Germ−/−^* testis), whereas the A_undiff_-A_diff_ transition indicating the onset of the second round was delayed by 3 days (i.e. it occurred at 9 DPS, instead of 6 DPS) (Tables 2 and 3). We assumed, in light of these observations, that the two transitions that mark off the spermatogonia differentiation process (A_undiff_-A_diff_ transition and entry into meiosis) may actually require different concentrations of ATRA. To test this hypothesis, we synchronized the spermatogenesis in control and *Aldh1a1-3^Germ−/−^* pups, and then treated them for 4 consecutive days spanning the A_undiff_-A_diff_ transition and entry into meiosis (Table 2) with WIN18,446, an inhibitor of RALDH (Arnold et al., 2015; Paik et al., 2014), or vehicle (DMSO). We analyzed their testes 1 day after the last injection, at 7 DPS (Fig. 8A). Treatment with WIN18,446 impaired the emergence of A_diff_ spermatogonia (compare Fig. 8D with E), but had no effect on the emergence of spermatocytes, which had reached the zygotene stage (SYCP3+), as in DMSO-treated testes (compare Fig. 8B with C). Moreover, expression of *Stra8* was present in spermatocytes, but not in spermatogonia exposed to WIN18,446 (compare Fig. 8D with E). This situation faithfully mimics the *Aldh1a1-3^Ser−/−^* phenotype at 7 and 8 DPS (Fig. 6; Table 3). We conclude that the concentrations of ATRA required for the A_undiff_-A_diff_ transition and expression of *Stra8* in spermatogonia are significantly higher than those needed to enter meiosis and express *Stra8* in spermatocytes.

### Removing both the Sertoli and germ cell-derived sources of ATRA definitively blocks spermatogonia differentiation

**Fig. 9.**
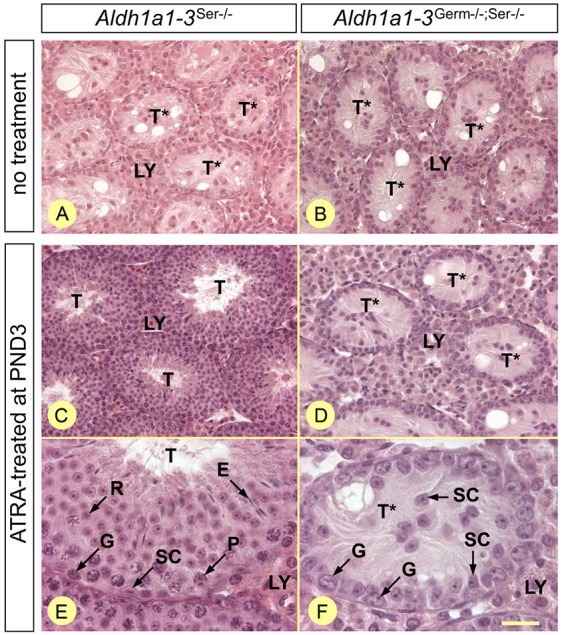
**Neonatal exposure of *Aldh1a1-3^Germ−/−;Ser−/−^* mutants to exogenous ATRA cannot sustain spermatogenesis.** (A,B) Histological sections of testes from untreated 6-week-old mutant males. (C-F) Histological sections 6 weeks after a single injection of ATRA at P3. Genotypes are as indicated. All sections were stained with Hematoxylin and Eosin. E, elongated spermatids; G, spermatogonia; LY, Leydig cells; P, pachytene spermatocytes; R, round spermatids; SC, Sertoli cells; T and T*, tubule cross-sections displaying spermatogenesis or only Sertoli cells and spermatogonia, respectively. Scale bar: 60 µm in A-D; 20 µm in E,F.

The SE of adult mice lacking RALDH both in GCs and SCs (*Aldh1a1-3^Germ−/−;Ser−/−^* compound mutants) analyzed at 6 and 30 weeks of age (*n*=3 mice at each time point) displayed only SCs and ZBTB16+, KIT−, STRA8− A_undiff_ spermatogonia (Fig. 9B; Fig. S10). This is similar to the situation described in *Aldh1a1-3^Ser−/−^* adults (Fig. 9A; Raverdeau et al., 2012). To determine whether exogenous ATRA could rescue spermatogenesis as in *Aldh1a1-3^Ser−/−^* mice (Raverdeau et al., 2012), we treated *Aldh1a1-3^Germ−/−;Ser−/−^* mutants at P3 and analyzed their testis 6 weeks later, i.e. after a complete spermatogenic cycle. *Aldh1a1-3^Ser−/−^* mutants were used as controls in this experiment. We found that spermatogonia were the only GCs present in the SE of the ATRA-treated *Aldh1a1-3^Germ−/−;Ser−/−^* compound mutants (Fig. 9D,F), whereas, in accordance with previous results (Raverdeau et al., 2012), spermatogenesis was complete in *Aldh1a1-3^Ser−/−^* mutants, yielding elongated, mature, spermatids (Fig. 9C,E).

**Fig. 10.**
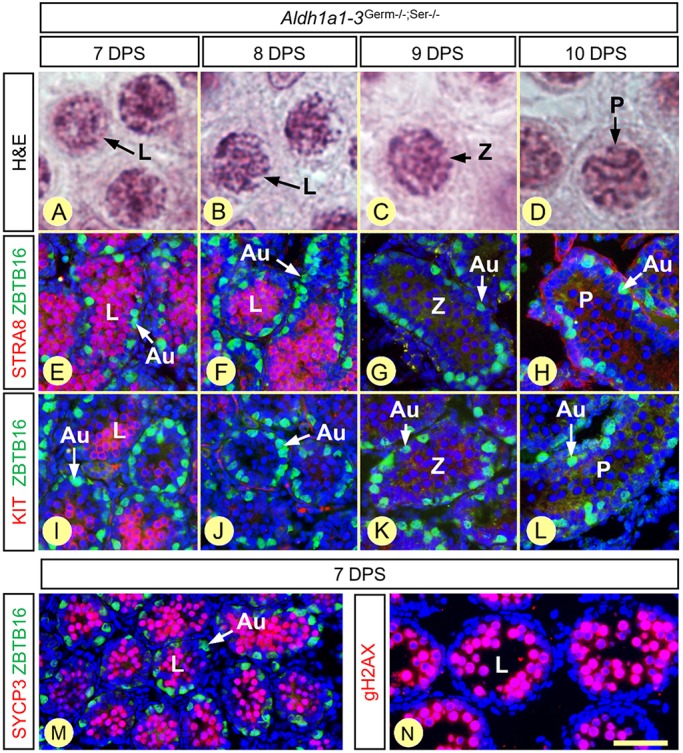
**Chronology of GC differentiation in *Aldh1a1-3^Germ−/−;Ser−/−^* testes at 7, 8, 9 and 10 days after synchronization.** (A-D) Morphology of the spermatocytes, present at the center of the seminiferous tubules, assessed by Hematoxylin and Eosin staining. (E-L) Characterization of the spermatogonia populations, present at the periphery of the seminiferous tubules, assessed by detection of ZBTB16 (green signal) and either STRA8 or KIT (red signals), as indicated. (M,N) Expression of the meiotic markers SYCP3 and gH2AX (red signals) in leptotene spermatocytes. Nuclei were counterstained with DAPI (blue signal). Au, A_undiff_ spermatogonia; DPS, day post-synchronization; L, Z and P indicate leptotene zygotene and pachytene spermatocytes, respectively. Scale bar: 6 µm in A-D; 60 µm in E-L; 80 µm in M; 40 µm in N.

We also studied the timeline of the first two rounds of spermatogonia differentiation in synchronized *Aldh1a1-3^Germ−/−;Ser−/−^* compound mutants at 7, 8, 9 and 10 DPS. The chronology of the first, ATRA-induced, round was similar to that of *Aldh1a1-3^Ser−/−^* mutants (Figs 6B-E and 10A-D; Tables 3 and 4). At 7 DPS, A_undiff_ spermatogonia yielded leptotene spermatocytes, which were indistinguishable from control spermatocytes in terms of expression of the early meiotic markers STRA8, SYCP3 and gH2AX (Fig. 10E,M,N) (Hamer et al., 2003; Mark et al., 2015; Yuan et al., 2000, and references therein). In contrast, A_undiff_ spermatogonia of the second round of differentiation did not progress further: they failed to express KIT and STRA8 at 9 DPS and stayed undifferentiated (ZBTB16+, KIT−, STRA8−) at 10 DPS (Fig. 10E-L; Table 4), as well as at later developmental stages (i.e. 12 and 14 DPS, not shown). Altogether, these data indicate, in *Aldh1a1-3^Germ−/−;Ser−/−^* compound mutant mice, that spermatogonia cannot differentiate unless exposed to exogenous ATRA. They also indicate that the spermatocytes originating from the spermatogonia exposed to ATRA at P3 initiate meiosis and express canonical meiotic markers, including STRA8, despite the lack of both the GC- and SC-derived sources of ATRA in the SE.

**Table 4.**
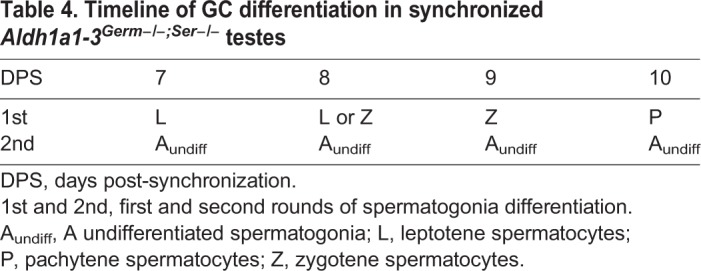
**Timeline of GC differentiation in synchronized *Aldh1a1-3^Germ−/−;Ser−/−^* testes**

## DISCUSSION

### Two local, RALDH-derived, sources of ATRA trigger and maintain the seminiferous epithelium cycle and wave

Our observations that the cycle and wave of the SE, as well as the expression patterns of GC-specific ATRA-responsive genes *Stra8* and *Rec8* (Koubova et al., 2014), appear fully normal in *Aldh1a1-3^Germ−/−^* mutants demonstrate that the SC-derived source of ATRA is sufficient to ensure a normal adult spermatogenesis in steady-state conditions. However, as assessed from the transcriptome analysis, which shows decreased expression of at least one gene required for optimal fertilization (i.e. *Lgals1*; Vasen et al., 2015), the GC source can be useful to strengthen the reproductive capacity. Most importantly, the inability to restore a sustainable spermatogenesis in *Aldh1a1-3^Germ−/−;Ser−/−^* compound mutants upon administration of ATRA unambiguously demonstrates that the SE is the only endogenous source of ATRA that allows for the transition from A_undiff_ to A_diff_ spermatogonia. It also proves that this source stems solely from RALDH activities and thus goes against the existence of alternative enzymatic activities for ATRA biosynthesis in the SE (Beedle et al., 2018). More specifically, the *Aldh1a1-3^Germ−/−;Ser−/−^* phenotype rules out the possibility that ATRA synthesized by Leydig cells, or any other cell type present in the interstitial spaces between seminiferous tubules, could trigger the A_undiff_-A_diff_ transition and thus initiate spermatogenesis. It also proves that the absence of reproductive defects in *Aldh1a1-3^Germ−/−^* mutants cannot be accounted for by the expression in spermatocytes of other enzymes endowed with the capacity to produce ATRA.

Surprisingly, RALDH activity either in GCs (present study) or in SCs (Raverdeau et al., 2012) is dispensable to the control of periodic gene expression in SCs (i.e. the SC cycle). In contrast, impairment of ATRA signaling in SCs either by deletion of *Rara* or by expression of a dominant-negative *Rara*, causes deregulation of the SC cycle (Hasegawa and Saga, 2012; Vernet et al., 2006a). These differences may originate from ATRA-independent RAR signaling, which are impaired in *Rara*-knockout mutants but not in RALDH-deficient mice.

Together, our observations demonstrate that the SC- and GC-derived sources of ATRA exert redundant functions in the maintenance of the adult SE cycle. They do not therefore support the scenarios according to which ATRA of spermatocyte origin is the driving force that allows the timely differentiation of A_undiff_ spermatogonia, which in turn initiates the SE cycle (Sugimoto et al., 2012).

### ATRA made by Sertoli and germ cells co-regulates A_undiff_-A_diff_ transitions during pubertal spermatogenesis

Spermatogonia, the only GC type present in the neonatal testis, cannot cell-autonomously trigger the A_undiff_-A_diff_ transition that initiates pubertal spermatogenesis because they are unable to produce ATRA (Kent et al., 2016; and present study). Actually, the only source of ATRA available to neonatal spermatogonia depends on RALDH activities in SCs (Raverdeau et al., 2012). Accordingly, the first A_undiff_-A_diff_ transition is unable to take place *Aldh1a1-3^Ser−/−^* mutants (Raverdeau et al., 2012), is delayed in *Aldh1a1^−/−^* mutants (Vernet et al., 2006a), and follows the same timing in *Aldh1a1-3^Germ−/−^* mutants and wild-type mice (present report). A_undiff_-A_diff_ transitions, which initiate sequential waves of complete spermatogenesis, continue for months in rescued *Aldh1a1-3^Ser−/−^* mutants. The RALDH activity required to sustain these transitions in *Aldh1a1-3^Ser−/−^* mutants is necessarily supported by their own progeny because ATRA synthesized outside the seminiferous cords cannot reach spermatogonia (Raverdeau et al., 2012; Teletin et al., 2017; Vernet et al., 2006b). We show here that RALDH2 activity in zygotene and more advanced spermatocytes is the only possible source of ATRA for perpetuating spermatogenesis in rescued *Aldh1a1-3^Ser−/−^* mutants, where it acts in a paracrine fashion to induce the A_undiff_-A_diff_ transition for the second and subsequent rounds of spermatogonia differentiation.

Whether ATRA could influence the duration of spermatogonia differentiation has not been investigated so far. Here, we found that only 6 days (versus 8.6 in the normal adult situation) space out the appearance of A_diff_ spermatogonia from that of preleptotene spermatocytes in synchronized neonatal testes. This is in agreement with previous studies showing that spermatogonia differentiation proceeds faster in prepubertal than in adult testes (Drumond et al., 2011; Kluin et al., 1982). In any event, the duration of spermatogonia differentiation is not dramatically influenced by the reduced ATRA concentrations in the SE, because 6 days after the appearance of A_diff_ spermatogonia, spermatocytes are at similar stages of maturation (i.e. preleptotene and/or leptotene) in *Aldh1a1-3^Germ−/−^* and *Aldh1a1-3^Ser−/−^* mutants (Tables 2 and 3; Fig. 5).

### ATRA of germ cell origin participates in the propagation of spermatogenesis along the seminiferous tubules

At the start of spermatogenesis during puberty, ATRA synthesized by RALDH1 and RALDH2 in SCs triggers the first A_undiff_-A_diff_ transitions (Snyder et al., 2011; Raverdeau et al., 2012). Several days later, spermatocytes start expressing RALDH2. From these new sources, ATRA can diffuse locally to recruit more A_undiff_ spermatogonia towards the differentiation pathway, thereby propagating the pubertal spermatogenic wave. The delayed pubertal spermatogenesis observed in *Aldh1a1-3^Germ−/−^* mutants supports the hypothesis that the GC-derived source of ATRA can increase the frequency of the A_undiff_-A_diff_ transition events in the SE.

Interestingly, the view that the ATRA signal can be transferred intratubularly from SCs to GCs and then propagated by the GCs themselves along the seminiferous tubules is supported by the patterns of spermatogenesis recovery in *Aldh1a1-3^Ser−/−^* mutants, where the only possible source of ATRA is represented by the epithelial cells of the rete testis (Fig. S11). Accordingly, in *Aldh1a1-3^Ser−/−^* mutants, spermatogenesis eventually reinitiates synchronously in seminiferous tubule segments closest to the rete testis and subsequently spreads thanks to the RALDH activity of GCs (Fig. S11). It is important to note, along these lines, that spontaneous re-initiation of spermatogenesis from the rete testis never occurs in *Aldh1a1-3^Germ−/−;Ser−/−^* compound mutants.

### ATRA acts in a non-cell-autonomous manner to promote the entry into meiosis

There is clear evidence from gene knockout studies that, in the male gonad, STRA8 plays indispensable functions in the switch from a mitotic to a meiotic mode of cell division (Anderson et al., 2008; Mark et al., 2008). In contrast, there is no general agreement about when, where, and to what extent ATRA is involved in the initiation of *Stra8* expression by spermatocytes and of male meiosis.

We have proposed that the source of ATRA allowing *Stra8* expression and meiotic initiation originates from preleptotene spermatocytes themselves (Raverdeau et al., 2012). The inability to detect the RALDH2 protein in these cells (Kent et al., 2016; present results) does not rule out the possibility that it may be expressed at very low levels, below the detection threshold of the antibody. However, the finding that meiosis occurs with a normal timing in spermatocytes lacking all RALDH (i.e. in *Aldh1a1-3^Germ−/−^* mutants) definitively rules out our hypothesis that physiological ATRA would initiate meiosis in spermatocytes through controlling *Stra8* expression in a cell-autonomous manner.

We have also shown that treatment of mice with the pan-RAR inverse agonist BMS-204,493 abrogates *Stra8* expression and induces premature condensation of chromosomes (a hallmark of the *Stra8^−/−^* phenotype), thereby blocking the onset of meiosis (Raverdeau et al., 2012). We concluded that ATRA was necessary to initiate this process. In contrast, our present finding indicates that meiosis is not blocked, but only slightly delayed, in mice lacking all RALDH in the SE: leptotene spermatocytes in *Aldh1a1-3^Ser−/−^* and *Aldh1a1-3^Germ−/−;Ser−/−^* mutants appear 24 h later than their counterparts in control mice, but they normally express STRA8 and subsequently progress to the pachytene stage. The strong effect of BMS-204,493 on STRA8 expression and meiotic initiation is probably due to its ability to stabilize the binding of RAR to co-repressor complexes that would cause transcriptional silencing at the *Stra8* locus through histone deacetylation and chromatin condensation (Germain et al., 2009). Actually, the results of the present genetic analysis demonstrate that, in the male GC lineage, ATRA is not decisive to meiotic initiation, but it merely facilitates this process. Our finding is somewhat reminiscent of the observation that, in the female GC lineage, meiosis also occurs normally in fetal gonads lacking ATRA as a result of RALDH2 and RALDH3 ablations (Kumar et al., 2011).

### A single threshold value of ATRA concentration cannot account for the coupling of spermatogonia and spermatocyte differentiation

In the adult testis, ATRA concentrations vary periodically in an epithelial stage-related manner. The highest level of ATRA, at stages VII-IX, coincides with A_undiff_-A_diff_ transition, meiotic initiation and expression of *Stra8* in GCs (Hogarth et al., 2015). These observations have led to the proposal that an ATRA-STRA8 signaling pathway acts instructively in both spermatogonia differentiation and meiotic initiation, causing these transitions to occur at the same time and place, precisely when GC competencies intersect with high ATRA levels in the SE (Endo et al., 2015). It has also been shown, in a mouse model of synchronized pubertal spermatogenesis, that the time points displaying highest ATRA concentrations correspond with the presence of STRA8-positive spermatogonia or preleptotene spermatocytes, or both (Hogarth et al., 2015).

We have found here that reducing the levels of ATRA by WIN18,446 treatment in testes that are already deprived of the GC-derived source of ATRA can arrest spermatogonia at the A_undiff_ stage without affecting the timing of the onset of meiosis. We additionally show in synchronized *Aldh1a1-3^Germ−/−;Ser−/−^* compound mutants that meiotic spermatocytes can emerge normally while, at the same time, differentiation of spermatogonia is totally arrested. These findings indicate that the concentration of ATRA required to enter meiosis is significantly below that triggering the A_undiff_-A_diff_ transition, not to say it is equal to zero. This is in keeping with our observations that arrested spermatogonia, but not arrested preleptotene spermatocytes nor spermatocytes presenting a *Stra8^−/−^* phenotype, are observed in the course of vitA-deficiency in the mouse (Ghyselinck et al., 2006). Altogether, these observations do not support the view that reaching a given threshold of ATRA concentration in the SE represents the cornerstone for coordinating the timing of the A_undiff_-A_diff_ transition with meiotic initiation.

### Spermiation requires ATRA synthesis in Sertoli cells

Spermiation is impaired in rodent models of vitA-deficiency and in rescued *Aldh1a1-3^Ser−/−^* mutants (Ghyselinck et al., 2006; Raverdeau et al., 2012). Our present observation that spermiation occurs normally, throughout life, in mice lacking all RALDH in GCs indicates that the SC-derived source of ATRA is sufficient to ensure normal spermiation. It does not support the view that the spermatocyte-derived source of ATRA is specifically required for this process (Endo et al., 2017).

In mice, RALDH2 contributes 61% of the total ATRA synthesis in the adult testis where, according to IHC and ISH data, its expression is restricted to GCs (Arnold et al., 2015; Vernet et al., 2006b; present results). Accordingly, the *Aldh1a1-3^Germ−/−^* mutation reduces by 63% the intratesticular ATRA concentrations. The ATRA remaining in the SE of *Aldh1a1-3^Germ−/−^* mutants (37%) is generated by RALDH activities located in cells other than GCs. RALDH3 is faintly expressed in Leydig cells only and RALDH4 is absent from the testis (Figs S2 and S9). Accordingly, RALDH1 activity is proposed to account at most for 39% of the ATRA in the whole adult testis (Arnold et al., 2015). Because (1) RALDH1 is not confined to SCs (Leydig cells also strongly express RALDH1) and is 15 times less efficient than RALDH2 at producing ATRA (Gagnon et al., 2002), and (2) SCs represent less than 20% of the volume of the murine SE (Russell, 1990), these data indicate together that the contribution of SCs to ATRA levels normally present in the SE is minimal, but is nonetheless essential for spermiation. These data suggest that, in the normal situation, ATRA must act within SCs to promote spermiation.

## MATERIALS AND METHODS

### Mice

Mice were on a mixed C57BL/6-129/Sv (50-50%) genetic background. They were housed in a licensed animal facility (agreement A67-218-37). All experiments were approved by the local ethical committee (Com'Eth, accreditations 2012-080 and 2012-081) and were supervised by N.V., N.B.G. or M.M., who are qualified in compliance with the European Community guidelines for laboratory animal care and use (2010/63/UE). To inactivate *Aldh1a*-coding genes in spermatogonia and their descendants, mice carrying *lox*P-flanked alleles (L2) of *Aldh1a1*, *Aldh1a2* and *Aldh1a3* (Raverdeau et al., 2012) were crossed with mice bearing the *Tg(Stra8-cre)^1Reb^* transgene (Sadate-Ngatchou et al., 2008). Cre-mediated ablation by *Tg(Stra8-cre)^1Reb^* transgene occurs in undifferentiated spermatogonia as early as P3 (Gely-Pernot et al., 2015; Sadate-Ngatchou et al., 2008). In F1, *Aldh1a1^L2/L2^;Aldh1a2^L2/L2^;Aldh1a3^L2/L2^* females were crossed with males bearing one copy of the transgene (*Stra8^tg/0^*). The resulting males (*Stra8-Cre^tg/0^;Aldh1a1^+/L2^;Aldh1a2^+/L2^;Aldh1a3^+/L2^*) were backcrossed with *Aldh1a1^L2/L2^;Aldh1a2^L2/L2^;Aldh1a3^L2/L2^* females to generate mutant males in F2 (*Stra8-Cre^tg/0^;Aldh1a1^L2/L2^;Aldh1a2^L2/L2^;Aldh1a3^L2/L2^*; referred to as *Aldh1a1-3^Germ−/−^* mutants), and their control littermates (*Aldh1a1^L2/L2^;Aldh1a2^L2/L2^;Aldh1a3^L2/ L2^* males). They did not display testis defects and were hereafter referred to as control mice. To generate *Aldh1a1^Ser−/−^* mutants or *Aldh1a1-3^Ser−/−^* mutants, mice carrying *loxP*-flanked alleles of *Aldh1a* genes were crossed with mice bearing the *Amh-Cre* transgene, as described previously (Raverdeau et al., 2012). To generate *Aldh1a1-3^Germ−/−;Ser−/−^* compound mutants, *Stra8-Cre^tg/0^;Aldh1a1^L2/L2^;Aldh1a2^L2/L2^;Aldh1a3^L2/L2^* males were crossed with *Amh-Cre^tg/0^;Aldh1a1^L2/L2^;Aldh1a2^L2/L2^;Aldh1a3^L2/L2^* females.

### Testis weight, seminiferous tubule diameters, and number of Sertoli and germ cells

Testes weights were determined from 9- to 10-week-old *Aldh1a1-3^Germ−/−^* mutants and control littermates (*n*=16 and 14, respectively). To measure seminiferous tubule diameters, transverse histological sections from 9- to 10-week-old *Aldh1a1-3^Germ−/−^* and control littermates (*n*= 4 mice in each genotype) were stained with periodic acid-Schiff (PAS) and scanned in a Hamamatsu NanoZoomer 2.0-HT: about 50 sections of seminiferous tubules that were nearly round were chosen randomly on each histological section and analyzed using the NDP*.*view*2* software. Germ cells were counted on the same histological sections, at stages VII and VIII for preleptotene and leptotene spermatocytes, and at stage X for early zygotene spermatocytes (on five tubule sections per animal and per stage). Sertoli cell nuclei were counted on sections from paraffin-embedded testes immunostained with an antibody directed against SOX9 (Table S1). The tubule circumference was measured in mm using the NDP*.*view*2* software and results were expressed as mean number of GC types/mm of tubule circumference±s.e.m. Statistical significance was assessed using Student's *t*-tests.

### Retinoid measurements

The retinoid contents were determined in testes from 9- to 10-week-old *Aldh1a1-3^Germ−/−^* and control littermates. Briefly, ATRA was quantified using liquid chromatography-tandem mass spectrometry (LC-MS/MS) on an AB Sciex 5500 QTRAP in multistage-MRM (multiple reaction monitoring) mode using APCI (atmospheric pressure chemical ionization) in positive ion mode, as previously described (Jones et al., 2015). Retinol and retinyl esters were quantified by high-performance liquid chromatography with ultraviolet detection (HPLC-UV) on a Waters ACQUITY system using a method that has been previously described (Kane et al., 2008). Retinoids are expressed in mol/g tissue. Statistical significance was assessed using Student's *t*-tests.

### Treatments

The onset of spermatogenesis normally occurs asynchronously at different sites of the SE. This generates extensive GC heterogeneity along the length of the testis tubules, which precludes the monitoring of cell differentiation. To overcome the population heterogeneity, the timeline of spermatogonia differentiation was monitored in a synchronous SE (Davis et al., 2013). Synchronization was induced, by injecting subcutaneously a single dose of ATRA (16 mg/kg; 50 µl of 1 mg/ml ATRA prepared in a sunflower oil/ethanol mixture, 9/1 v/v) at P3. The ‘synchronized’ testes samples were dissected after 24 h, then at intervals of 1 day, starting 5 days and ending 12 or 14 days following the synchronization treatment (days post-synchronization, DPS). In one experiment aimed at investigating the effects of pharmacological inhibition of RALDH on spermatogenesis, mice were first injected with ATRA at P3, treated intraperitoneally with either 25 mg/kg/day WIN18,446 or vehicle (DMSO) for 4 days at 3, 4, 5 and 6 DPS, and then euthanized at 7 DPS, i.e. 24 h after the last injection.

Synchronized testes from at least three mice per genotypes and treatments were collected at each time-point. One testis, destined for immunohistochemical detection of ZBTB16, STRA8, KIT, SYCP3 and/or gH2AX was fixed in 4% (w/v) buffered paraformaldehyde (PFA), then embedded in freezing medium. The contralateral testis was either fixed in Bouin's fluid, then embedded in paraffin wax for histological analyses, or fixed in 4% PFA, then embedded in paraffin wax for detection of RALDH, DDX4 or GFP.

### Immunohistochemistry procedures

Antibodies used in immunohistochemistry (IHC) experiments are listed in Table S1. Histological sections were rinsed in phosphate-buffered saline (PBS), then incubated with appropriate dilutions of the primary antibodies or a mixture of them (i.e. anti-DDX4 and either anti-GFP or anti-SOX9, and anti-ZBTB16 and either anti-KIT or anti-STRA8) in PBS containing 0.1% (v/v) Tween 20 (PBST) for 16 h at 4°C in a humidified chamber. After rinsing in PBST (three times for 3 min each), detection of the bound primary antibodies was achieved for 45 min at 20°C in a humidified chamber using: a Cy^TM3^-conjugated donkey anti-rabbit IgG, a Cy^TM3^-conjugated goat anti-rat IgG, a Cy^TM3^-conjugated goat anti-mouse IgG, an Alexa Fluor 488-conjugated donkey anti-goat, or an Alexa Fluor 488-conjugated goat anti-chicken IgG, depending on the origin of the primary antibody. Nuclei were counterstained with 4′,6-diamidino-2-phenyl-indole (DAPI) diluted at 10 µg/ml in the mounting medium (Vectashield; Vector). Each experiment was repeated twice on at least three different mice per genotype, age and treated group. Owing to the high sequence similarities between RALDH isotypes, the specificity of the antibodies used to detect these proteins was carefully checked (Figs S1 and S2)

### Characterization of GC subtypes in ‘synchronized’ prepubertal testes

As morphological differences between spermatogonia types are both subtle and variable (Chiarini-Garcia and Russell, 2001), we chose to characterize them by labeling with antibodies against protein markers that have been linked to cell fate or function: ZBTB16, which is required for the maintenance of the spermatogonia stem cell pool (Buaas et al., 2004; Costoya et al., 2004); and STRA8 and KIT, which are typical makers of the transition from A_undiff_ to A_diff_ spermatogonia (Blume-Jensen et al., 2000; Evans et al., 2014; Kissel et al., 2000; Schrans-Stassen et al., 1999). We based our classification on immunohistochemical data from adult testes (Fig. S3; Teletin et al., 2017), where stages of spermatogonia differentiation have been precisely correlated with stages of the SE cycle. Spermatocytes were characterized based on their nuclear morphologies (Russell, 1990), and on the expression of STRA8 and gH2AX (Figs S3 and S7).

### Characterization and counts of the GC associations in untreated testes

#### Prepubertal testes

*Aldh1a1-3^Germ−/−^*, *Aldh1a1^Ser−/−^* and control testes at P21 (*n*=3 for each genotype) were fixed in Bouin's fluid and a 5 µm transverse histological section was stained with Hematoxylin and Eosin and scanned in a Hamamatsu NanoZoomer 2.0-HT. Cross-sections of seminiferous tubules (*n*>200 per histological section) were analyzed on the images using the NDP*.*view*2* software, and classified according to both the most advanced GC-type found near the lumen and to the GC type that formed the peripheral layer. Statistical analysis was carried out using a two-tailed Student's *t*-test, assuming unequal variances after arcsine transformation of the percentages of tubule sections containing at least two generations of meiotic and/or post-meiotic cells, as described previously (McDonald, 2014; Vernet et al., 2014).

#### Adult testes

The cellular composition of the epithelial stages was determined in *Aldh1a1-3^Germ−/−^* testes at 8-10 weeks (*n*=5), 12 months (*n*=2) and 18 months (*n*=1) of age. Testes were either fixed in Bouin's fluid or in 4% (w/v) PFA and embedded in paraffin wax. Transverse histological sections (5 µm) were then stained with periodic acid-Schiff (PAS) and counterstained with Hematoxylin (in the case of Bouin's-fixed samples) or with Alexa Fluor 488-conjugated peanut agglutinin (PNA, lectin from Arachis hypogaea), and counterstained with DAPI (in the case of PFA fixed samples). The most important identifying features were as follow: stages I-III, proacrosomal granule small and unstained or stained weakly by PAS or PNA; stage IV, acrosomic granule forming an indentation of the round spermatid nucleus and beginning to flatten; stages V-VI, acrosomic system forming a straight PAS- or PNA-positive line, the angle subtended by the acrosome being fewer or equal to 120°; stages VII-VIII, acrosome forming a cap that covers more than one-third of the round spermatid nucleus; stage IX, oblong spermatid nuclei; stage X, elongating spermatid head displaying a sharp angle between its ventral and caudal surface; stage XI, spermatid nucleus thinner, more elongated and stained more intensely; and stage XII, presence of meiotic metaphases, meiotic anaphases and/or secondary spermatocytes. The determination of epithelial stage frequencies was performed on four *Aldh1a1-3^Germ−/−^* mutants and 10 control mice at 9-10 weeks of age. A transverse histological section through the middle of each testis sample was stained with PAS, then scanned and the tubule cross-sections (>200 per sample) were analyzed. There were no significant differences in the distribution of stages over the tubule cross-sections in the 10 controls (χ^2^=73 with 63° of freedom; *P*=0.18) or in the four mutants (χ^2^=32 with 21° of freedom; *P*=0.06), indicating the two groups are homogeneous. Then, the synchronization factors were determined using the method described by van Beek and Meistrich (1990), and compared using a two-tailed Student's *t*-test assuming unequal variances.

### Transcriptome

Total RNA was prepared using TRIzol reagent (Life Technologies). Biotinylated single strand cDNA targets were prepared, starting from 250 ng of total RNA, using the GeneChip WT PLUS Reagent Kit (ThermoFisher Scientific, P/N 902280) according to the manufacturer's recommendations. Following fragmentation and end-labeling, 2 μg of cDNAs were hybridized for 16 h at 45°C on Mouse Clariom S arrays (Affymetrix), interrogating over 22,000 well-annotated RefSeq genes. The chips were washed and stained in the GeneChip Fluidics Station 450 (Affymetrix) and scanned with the GeneChip Scanner 30007G (Affymetrix) at a resolution of 0.7 µm. Raw data (.CEL Intensity files) were extracted from the scanned images using the Affymetrix GeneChip Command Console (AGCC) version 4.1.2. CEL files were further processed with Affymetrix Expression Console software version 1.4.1 to calculate probe set signal intensities using Signal Space Transformation-Robust Multi-array Average (SST-RMA) algorithms with default settings. The data discussed in this publication (Edgar et al., 2002) are available in GEO under accession number GSE121326 (www.ncbi.nlm.nih.gov/geo/query/acc.cgi?acc=GSE121326).

### Real-time quantitative RT-PCR

Reverse transcription of total RNA followed by PCR amplification of cDNA (RT-PCR) was performed using QuantiTect Reverse Transcription (Qiagen) and LightCycler 480 SYBR Green I Master (Roche Diagnostics) kits, respectively. Primers were as indicated in Table S2. Triplicates of at least three samples were used in each experimental condition. The transcript levels were normalized relative to those of *Gapdh* transcripts, the expression of which is not changed by the ablation of *Aldh1a* genes in GCs. Data were expressed as fold-induction relative to vehicle or control conditions. Statistical significance was assessed using Student's *t*-tests or by one-way ANOVA followed by the post-hoc Newman-Keuls test for comparison by pairs.

### *Aldh8a1* knockout

Mice carrying a targeted deletion of RALDH4 (*Aldh8a1* gene) were generated and analyzed as described in supplementary Materials and Methods.

## Supplementary Material

Supplementary information
