# Supplementary material for: Two functionally redundant sources of retinoic acid secure spermatogonia differentiation in the seminiferous epithelium
Source: Development. 2019 Jan 4;146(1):dev170225. doi: 10.1242/dev.170225 (PMC6340151; doi:10.1242/dev.170225)
Supplement: Supplementary information [file develop-146-170225-s1.pdf]

Antibodies	Origin	IHC protocol	Working dilution	Source	Reference
AR	RP	**1**	1/100	Santa Cruz	SC-816
CLDN3	RP	**1**	1/400	Invitrogen	341700
DDX4	RP	**1** or **2**	1/500	Abcam	ab13840
GFP	C	**1**	1/500	Aves	GFP-1020
KIT	RM	**2**	1/100	Cell Signaling	mAb #3074 (D13A2)
GATA1	RaM (N6)	**1**	1/50	Santa Cruz	SC-265
gH2AX	MM	**1** or **2**	1/500	Upstate	05-636 (JBW301)
RALDH2	RP	**1**	1/200	Abcam	ab75674
RALDH1	RM	**1**	1/50	Abcam	ab52492 (EP1933Y)
RALDH3	RP	**1**	1/20	Sigma	HPA046271
RALDH4	RP	**1**	1/100	Sigma	HPA055414
REC8	RM	**1**	1/200	Abcam	ab192241 (EPR16189)
SOX9	RP	**1** or **2**	1/100	Merck	Ab5535
STRA6	RP	**3**	1/1000	Bouillet et al., 1997	
STRA8	RP	**1**	1/500	Abcam	ab49602
STRA8	RP	**2**	1/1000	Oulad-Abdelghani et al., 1996	
SYCP3	RP	**2**	1/1000	Abcam	ab150292
ZBTB16	GP	**2**	1/500	R&D Systems	AF2944

Table S1. Antibodies used in IHC experiments. gH2AX, phosphorylated histone H2AX; C, chicken IgY fraction; GP, goat polyclonal; MM, mouse monoclonal; RM, rabbit monoclonal; RaM, rat monoclonal; RP, rabbit polyclonal; Testes were fixed in 4% (w/v) PFA in PBS for 16 h at 4°C, and washed in PBS. In adult mice, the fixative was first delivered by intracardiac perfusion. IHC protocol 1. Testes were embedded in paraffin. IHC was performed on 5 µm-thick dewaxed histological sections, after heat-induced antigen retrieval either in 10 mM citrate buffer pH 6.0 (in the case of IHC for detection of proteins other than REC8) or in Tris-EDTA at pH 9.0 [10 mM Tris Base, 1 mM EDTA, 0.05% (v/v) Tween 20] for 1 h in a water bath heated at 95°C (in the case of IHC for REC8). IHC protocol 2. Testes were cryoprotected in sucrose solutions and embedded in freezing medium. Cryosections, 10 µm-thick, were air-dried, hydrated in PBS and processed for IHC. IHC protocol 3.Testes were cryoprotected in sucrose solutions and embedded in freezing medium. Cryosections, 10 µm-thick, were air-dried, treated with cold acetone at - 20°C for 5 min, air dried again, then hydrated in PBS and processed for IHC. The figure in bold in the column “IHC protocol” refers to results illustrated in this study.

Gene	Accession number	Primers	Position (nt)	Product size (nt)
Aldh1a2	NM_009022.4	5'-AAGACACGAGCCCATTGGAG-3'<br>5'-GGAAAGCCAGCCTCCTTGAT-3'	574-593<br>734-753	180
Arhgap30	NM_001005508.2	5'-CCAGGACATTCACTGCGTCT-3'<br>5'-ACCAAGTGGCGCATAAGGAA-3'	559-578<br>746-765	207
Gapdh	NM_001289726.1	5'-CAAGGTCATCCATGACAACTT-3'<br>5'-GGCCATCCACAGTCTTCTGG-3'	569-589<br>638-657	89
Gm20877	NM_001199332.1	5'-TAATGTGCCTGCCACCCATT-3'<br>5'-CTCAGAGCCATGTTTCCTCTCA-3'	494-513<br>722-743	250
Lgals1	NM_008495.2	5'-GTCGCCAGCAACCTGAATCT-3'<br>5'-GGGCATTGAAGCGAGGATTG-3'	87-106<br>209-228	142
Lrif1	NM_001039488.1	5'-ACTATCTCCCGTCACACCCT-3'<br>5'-CGCTGCCAATCGTAGGAACT-3'	232-251<br>450-469	238
Olfr788	NM_146551.1	5'-GCCCCTTCACTACACAACCA-3'<br>5'-GCAAGACAGTTGCAAGAGGG-3'	375-394<br>539-558	184
Stra8	NM_009292.2	5'-CAACCTAAGGAAGGCAGTTTAC-3'<br>5'-GACCTCCTCTAAGCTGTTGGG-3'	256-278<br>408-428	173
Scp2	NM_011327.4	5'-TGTAGGAGACCTTCGCTGCT-3'<br>5'-TCCACTGCGGAGTAAGGGAT-3'	75-94<br>254-273	199
Vmn1r181	NM_207546.2	5'-ATGGACAATAGAACAGACTCCT-3'<br>5'-GGCCTCTGTTTAGAACCAGTCA-3'	74-96<br>279-300	227

Table S2. Primers used for quantification of mRNA levels by real-time RT-qPCR. Gene names, accession numbers, forward (upper line) and reverse (lower line) primers, their positions in the sequences and sizes of the amplified fragments are indicated. nt: nucleotide.

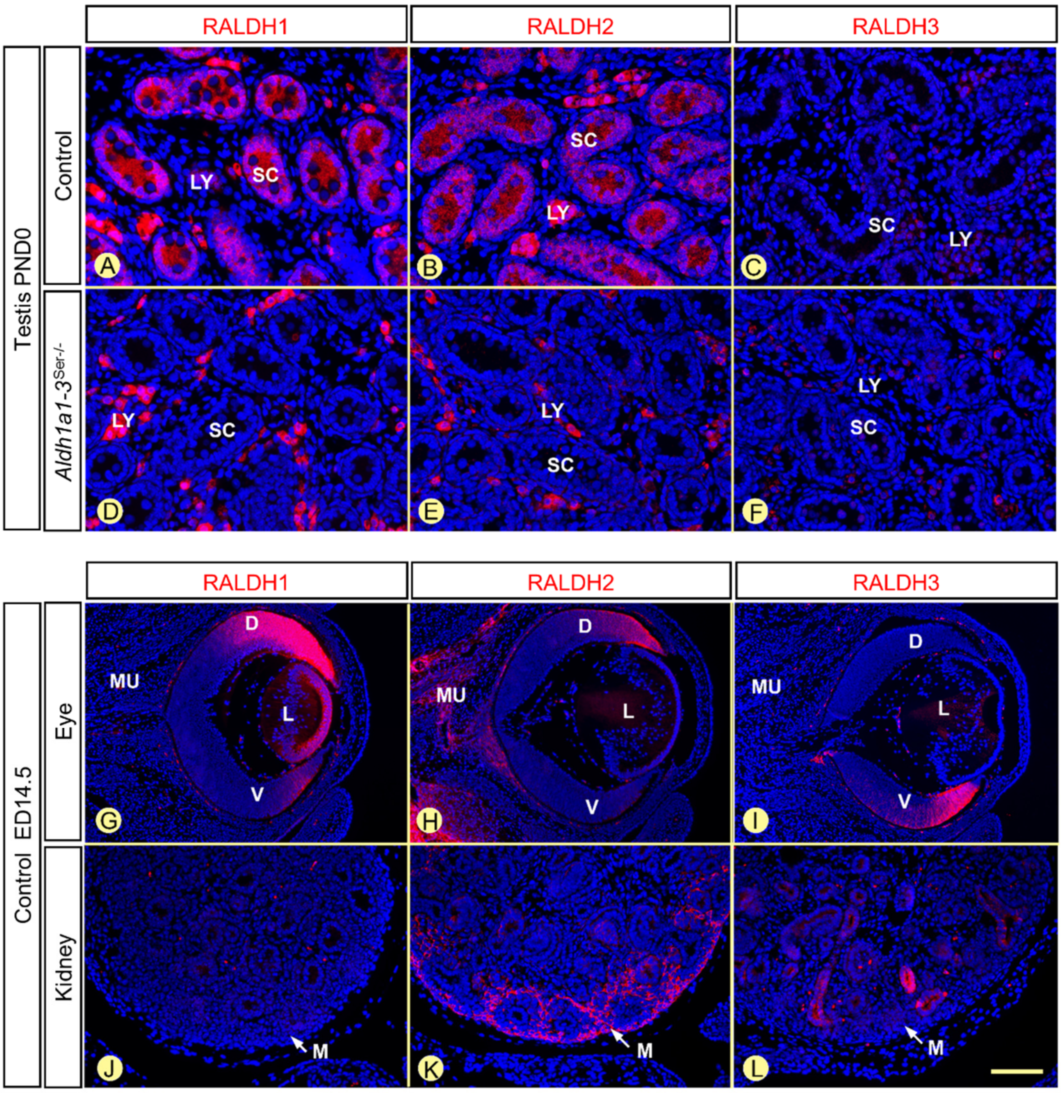

Fig. S1. Specificity of the antibodies directed against RALDH isotypes. Histological sections though the testes of wild-type (control) (A-C) and *Aldh1a1-3*$^{Ser-/-}$ (D-F) mice at PND0 or through the eye (G-I) and kidney (J-L) of control fetuses at embryonic day 14.5 (ED14.5), immunostained with antibodies to RALDH isotypes, as indicated (red signals). (A-F) Negative control experiment: RALDH1 and RALDH2 antibodies react with the cytoplasm of Sertoli cells (SC) in control (A,B), but not in *Aldh1a1-3*$^{Ser-/-}$ (D,E) testes, while RALDH3 is not detected (C,F). The RALDH2 signal in Leydig cells (LY) is a technical artifact, because *Aldh1a2* transcripts are absent from these cells (Vernet et al., 2006). (G-L) Positive control experiment: (G-I) The localization of RALDH1 in the dorsal retina (D), ventral retina (V) and lens (L), the localization of RALDH2 in the ocular muscles (MU), as well as the localization of RALDH3 in the ventral retina were expected from previous ISH data (Matt et al., 2005). In contrast, the signal generated by the anti-RALDH2 antibody at the tip of the dorsal retina, which does not express *Aldh1a2* transcripts (Matt et al., 2005), is a technical artefact that coincides with an area displaying high levels of RALDH1. Thus, the antibody to RALDH2 weakly cross-reacts with the RALDH1 antigen, but not with the RALDH3 antigen. It is however noteworthy that tissues where the levels of RALDH1 are lower, such as the lens, are not immunostained by the anti-RALDH2 antibody. (J-L) The mesenchyme of the kidney cortex (M), which represents one of the many fetal tissues displaying high levels of RALDH2, does not react with the anti-RALDH1 and anti-RALDH3 antibodies. Thus, antibodies to RALDH1 and RALDH3 do not cross-reacts with the RALDH2 antigen. Cell nuclei are stained by DAPI (blue signal). Scale bar (L): 60 µm (A-F), 160 µm (G-I) and 80 µm (J-L).

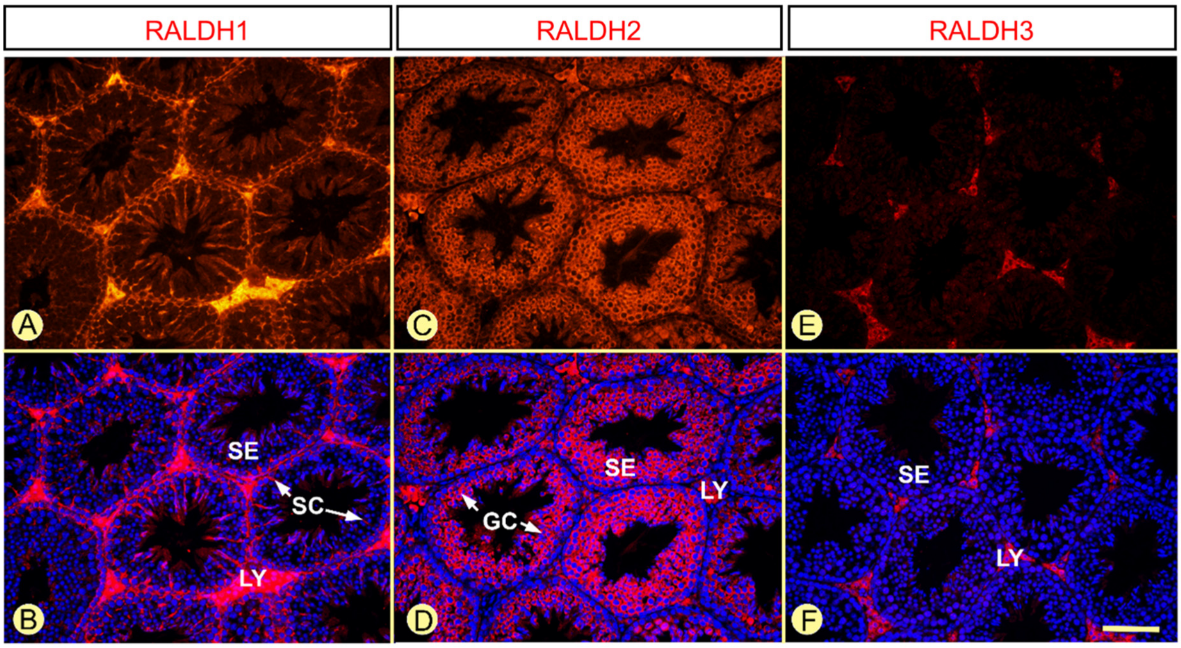

Fig. S2. Immunodetection of RALDH1, RALDH2 and RALDH3 (red signals) in wild-type adult testes. (A,B) RALDH1 is the main RALDH present in Sertoli cells (SC). (C,D) RALDH2 is the only RALDH detected in germ cells (GC). (E,F) RALDH3 is absent from the seminiferous epithelium (SE). Note that the RALDH2 signal in Leydig cells (LY) is a technical artifact, as *Aldh1a2* transcripts are absent from these cells (Vernet et al., 2006). Cell nuclei are stained by DAPI (blue signal in B,D,F). Scale bar (F): 80 µm (A-F).

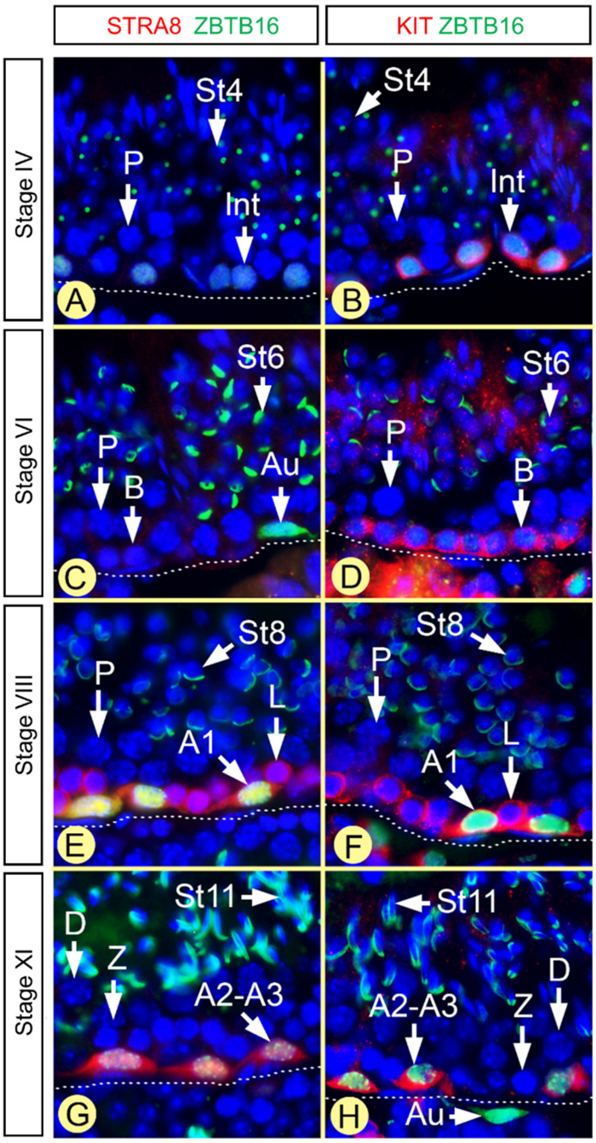

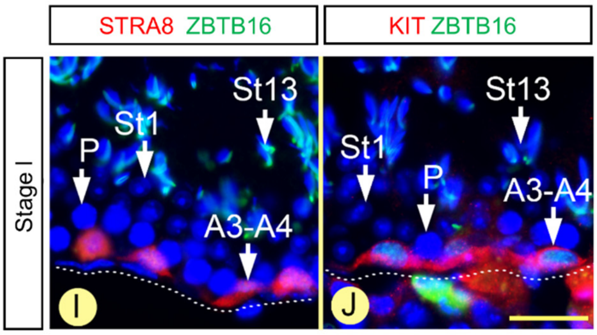

K

		ZBTB16	KIT	STRA8
II-IV	Int-B	+	++	-
	P	-	-	-
V-VI	B	-	++	-
	P	-	-	-
VII	A1	++	++	++
	prL	-	++	++
VIII	A1	++	++	++
	prL/L	-	++	++
IX	A1-A2	++	++	++
	L	-	++	++
	P	-	-	-
X	A2	++	++	++
	L/Z	-	-	+
XI-XII	A2-A3	++	++	++
	Z	-	-	-
I	A3-A4	++	++	+/++
	P	-	-	-

Fig. S3. Immunohistochemical classification of spermatogonia and spermatocytes present in the adult mouse testis. (A-J) are representative histological sections from double labelling experiment using anti-ZBTB16 antibodies (green signals) and either anti-KIT or anti-STRA8 (red signals) antibodies. Nuclei were counterstained DAPI (blue) and the acrosomal system with Alexa Fluor 488-conjugated peanut agglutinin (green). See Suppl. Table 1, IHC protocol 2 for technical details**.** (K) is a summary table of the expression of ZBTB16, KIT and STRA8 during the SE cycle (adapted from Ahmed and de Rooij, 2009). A_{undiff} spermatogonia, which are present at all stages of the SE cycle, are strongly labelled with antibodies to ZBTB16, but they do not express KIT nor STRA8. A_{undiff} spermatogonia are thus, phenotypically speaking, positive for ZBTB16 and negative for KIT and STRA8 (A_{undiff} phenotype: ZBTB16+;STRA8-;KIT-). A_{diff} spermatogonia, which include A1, A2, A3 and A4 spermatogonia, express, in addition to ZBTB16, STRA8 and KIT (A_{diff} phenotype: ZBTB16+;STRA8+;KIT+). Intermediate and B spermatogonia, in contrast to their A_{diff} precursors, express ZBTB16 at low to undetectable levels and are no longer positive for STRA8 (Int-B phenotype: ZBTB16-;STRA8-;KIT+). Also note that expression of STRA8 allows distinguishing preleptotene spermatocytes (ZBTB16-;STRA8+;KIT+) from their immediate precursor cells, B spermatogonia, which do not express STRA8 (ZBTB16-;STRA8-;KIT+; see also Vernet et al., 2006; Mark et al., 2015). ++ and + indicate robust and faint immunohistochemical signals, respectively. Abbreviations: Au, A1, A2, A3, A4, Int and B: spermatogonia; prL, L, P and Z: preleptotene, leptotene, pachytene and zygotene spermatocytes; St1 to St16: steps of spermatid maturation; S: Sertoli cells. Roman numerals designate the stages of the SE cycle. Scale bar (J): 30 µm (A-J).

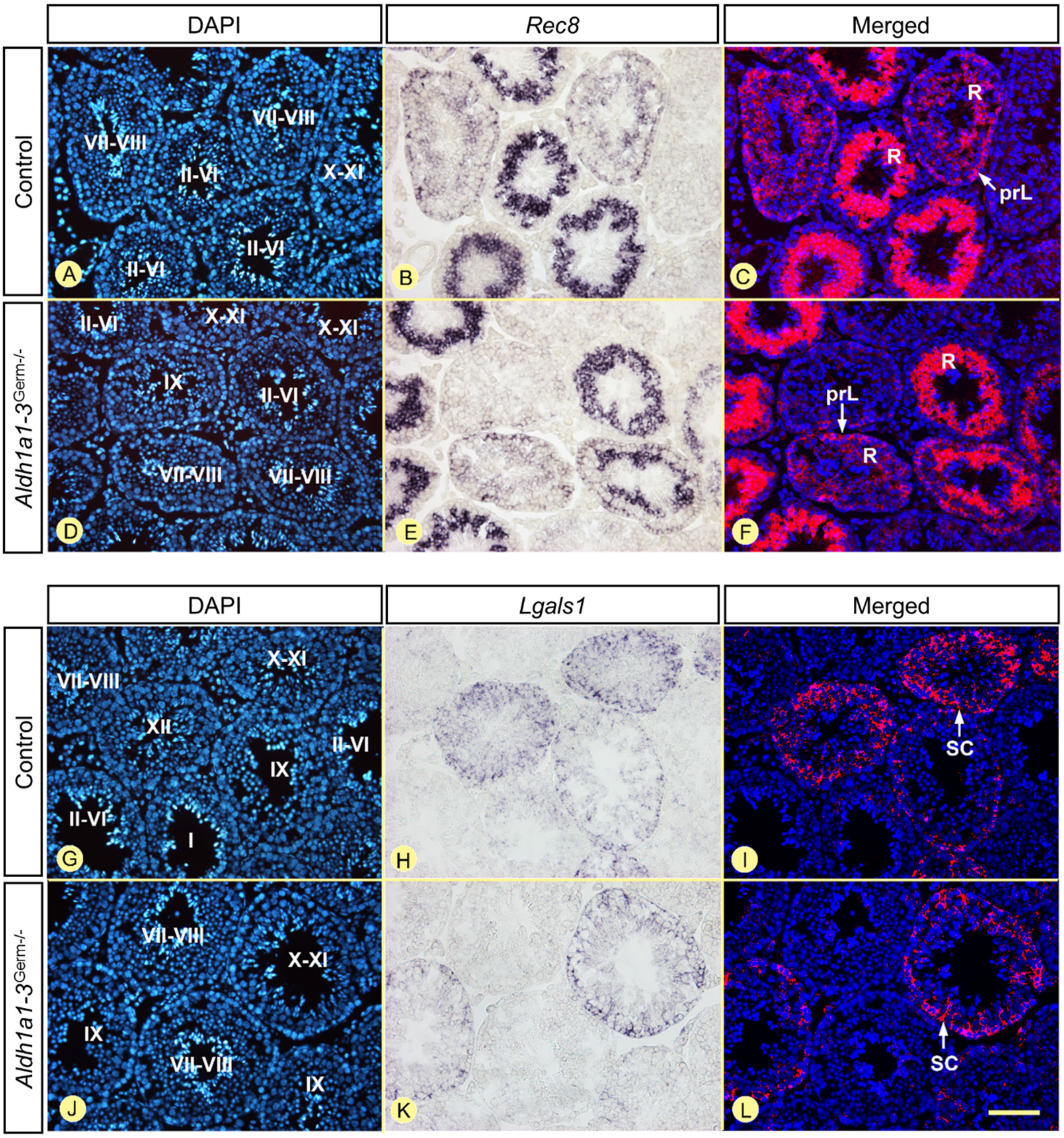

Fig. S4. Adult testes of control and *Aldh1a1-3*$^{Germ-/-}$ males display identical, epithelial stage-specific, distribution of *Rec8* and *Lgals1* transcripts. (A-F) In situ hybridization (ISH) using a *Rec8* antisense probe (purple signal in B,E). *Rec8* transcripts are expressed in preleptotene spermatocytes at epithelial stages VII and VIII. In round spermatids, *Rec8* transcripts are abundant in spermatids at stages II-VI and are absent from elongating spermatids at stages IX to XI. (G-L) ISH using an *Lgals1* antisense probe (purple signal in H,K). *Lgals1* transcripts are absent at stages II-VIII, peak at stages X-XII in Sertoli cells (SC), and are less abundant at stages IX and I. Cell nuclei are stained by DAPI (blue signal in A,D,G,J). ISH signals were converted to a red false color and superimposed with the DAPI nuclear counterstain (in C,F,I,L). prL, preleptotene spermatocytes; R, round spermatids; SC, Sertoli cells. Roman numerals designate the stages of the SE cycle: II–VI, stages II, III, IV, V, or VI; VII–VIII, stages VII or VIII; X-XI, stages X or XI. Scale bar (L): 80 µm (A-L).

Development • Supplementary information

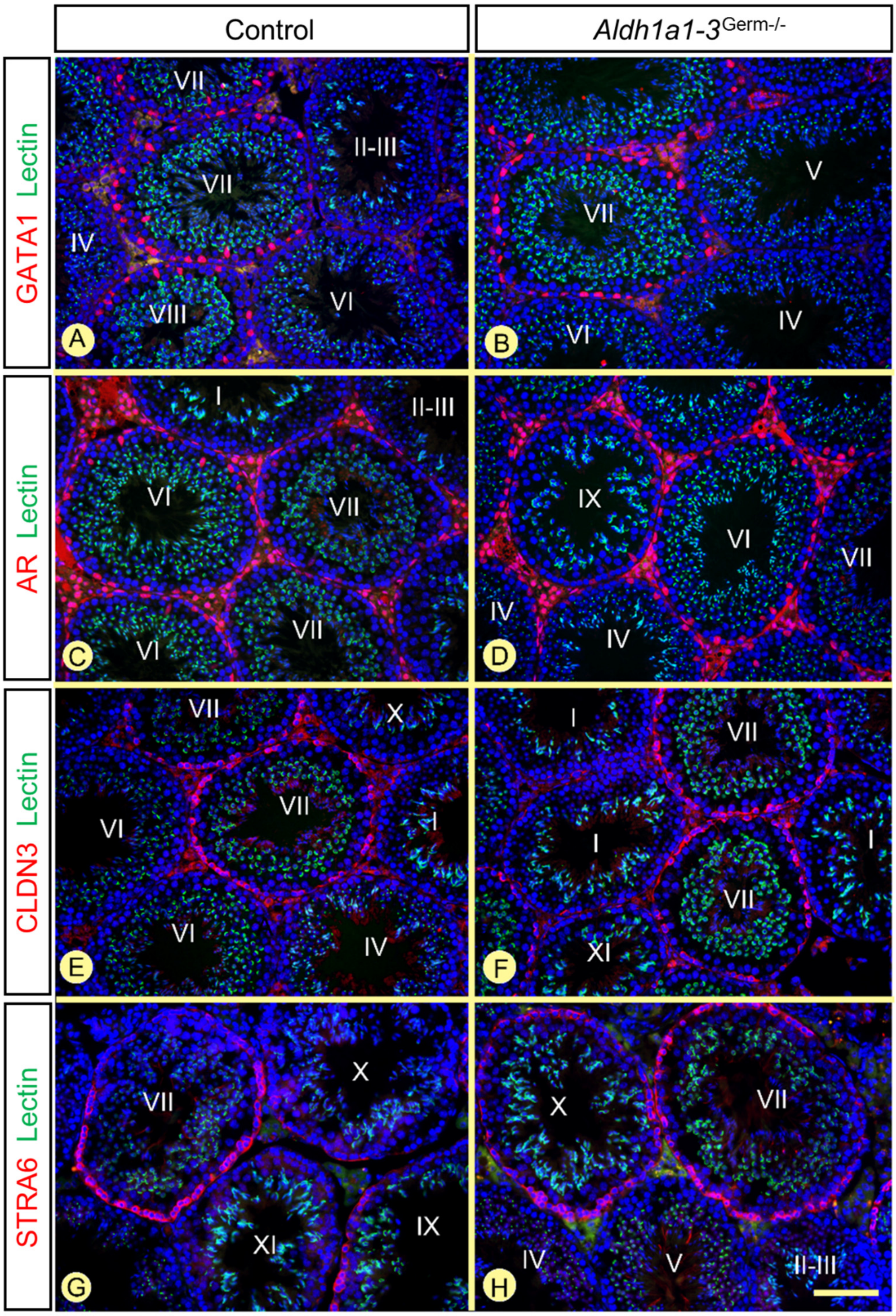

Fig. S5. Adult testes of control and *Aldh1a1-3*$^{Germ-/-}$ mice display identical, epithelial stage specific, distribution of GATA1, AR, CLDN3 and STRA6. Detection of AR, GATA1, STRA6 and CLDN3 (red signals) in control (A,C,E,G) and *Aldh1a1-3*$^{Germ-/-}$ (B,D,F,H) adult testes. AR is expressed in SC nuclei at all epithelial stages, with a conspicuous peak at stages VI and VII. GATA1 expression in Sertoli cell nuclei is undetectable at stages II-VI, weak at stages IX-XII and I and peaks at stages VII and VIII. Expressions of STRA6 and CLDN3, at the basolateral surface of SC, are undetectable at stages I-VI and XII, peak at stages VII-IX and decrease during stages X and XI. Nuclei were counterstained DAPI (blue signal) and the acrosomal system with Alexa Fluor 488-conjugated peanut agglutinin (green signal). Roman numerals designate the stages of the SE cycle. Scale bar (H): 80 μm (A-H).

Development • Supplementary information

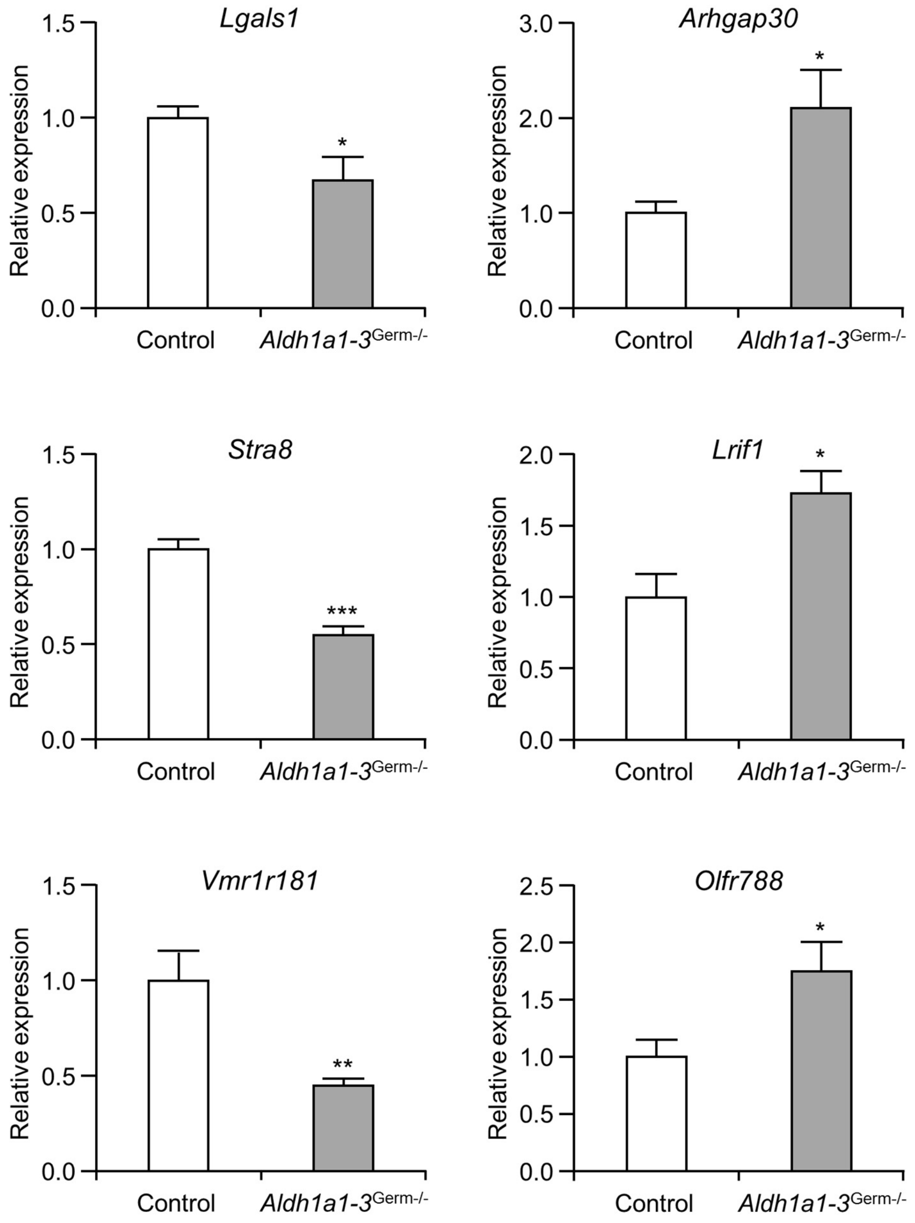

Fig. S6. Validation of the microarray results. The expression of six genes showing decreased (*Lgals1*, *Stra8*, *Vmr1r181*) or increased (*Arhgap30*, *Lrif1*, *Olfr788*) expression in *Aldh1a1-3*$^{\text{Germ-/-}}$ transcriptome were quantified by real-time RT-PCR using total RNA extracted from 9-10 weeks-old control (n=5) and *Aldh1a1-3*$^{\text{Germ-/-}}$ testes (n=5). *Gapdh* was used as internal control. Error bars indicate SEM ; *, *p*<0.05 ; **, p<0.01 and ***, p<0.001.

Development • Supplementary information

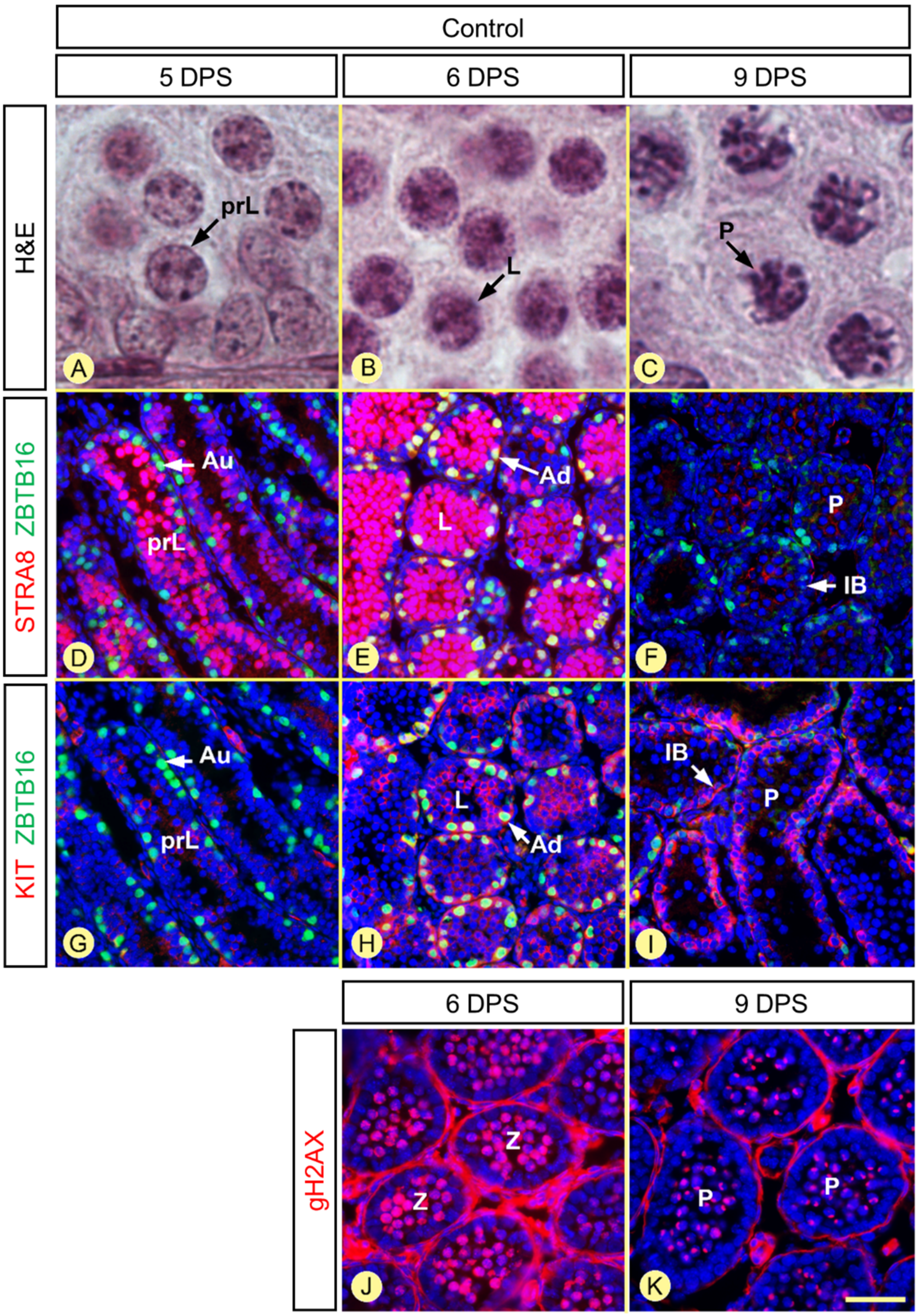

Fig. S7. Chronology of germ cell differentiation in control testes at 5, 6, 8 and 9 days after synchronization. (A-C) Morphology of the spermatocytes, present at the center of the seminiferous tubules, assessed by staining with hematoxylin and eosin staining (H&E). (D-I) Characterization of the spermatogonia populations, present at the periphery of the seminiferous tubules, by IHC staining with antibodies to ZBTB16 (green signal) and antibodies to either STRA8 or KIT (red signals), as indicated. (J-K) Expression of phosphorylated H2AX (gH2AX) allows distinguishing zygotene spermatocytes, in which the IHC signal is scattered within the nuclei, from pachytene spermatocytes, in which the signal is restricted to the XY body (Hamer et al., 2003). Nuclei were counterstained with DAPI (blue signal). Note that (D,G) and (E,H) are adjacent histological sections. Au, Ad and IB: A_{undiff}, A_{diff} and Intermediate or B spermatogonia, respectively; DPS, day post synchronization; prL, L, P and Z: preleptotene, leptotene, pachytene and zygotene spermatocytes, respectively. Scale bar (K) = 6 µm (A-C) and 60 µm (D-K).

Development • Supplementary information

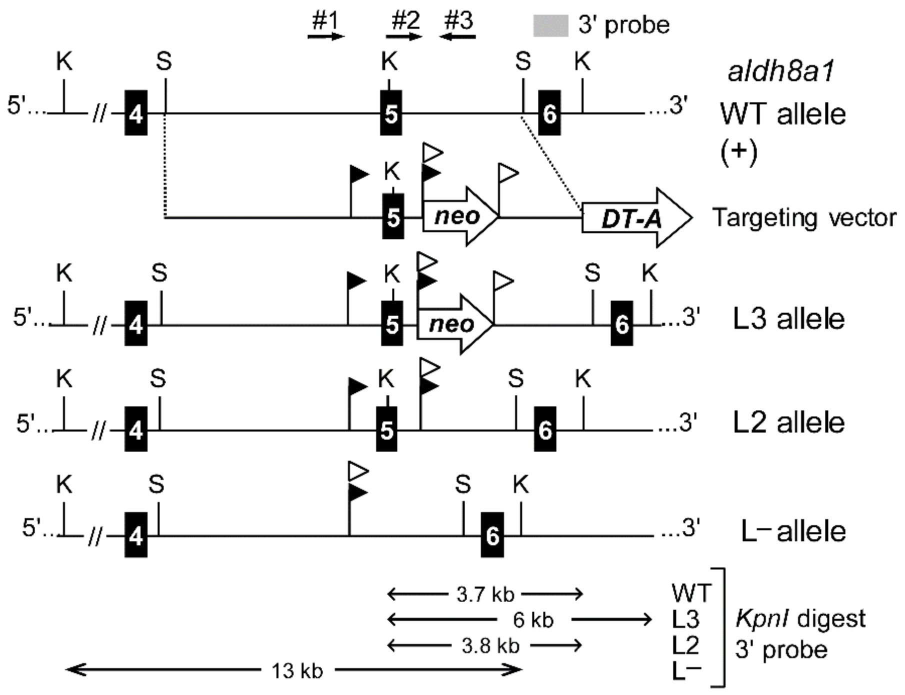

Fig. S8. Targeted disruption of the *Aldh8a1* gene encoding RALDH4. Structure of the targeting vector and partial restriction map of the *Aldh8a1* locus before [wild-type (WT) allele, +] and after homologous recombination (L3 allele) and Cre-mediated recombination (L2 and L- alleles). Black boxes (labeled 4-6) stand for exons. The location of restriction sites (K, *Kpn*I; S, *Spe*I) and of the 3' external probe is indicated. Black arrowhead flags represent loxP sites, while white arrowhead flags represent FRT sites. Arrows indicate the location of primers #1, #2 and #3 used for PCR genotyping. Sizes of the restriction fragments obtained for each allele are shown below and are in kilobases (kb).

Development • Supplementary information

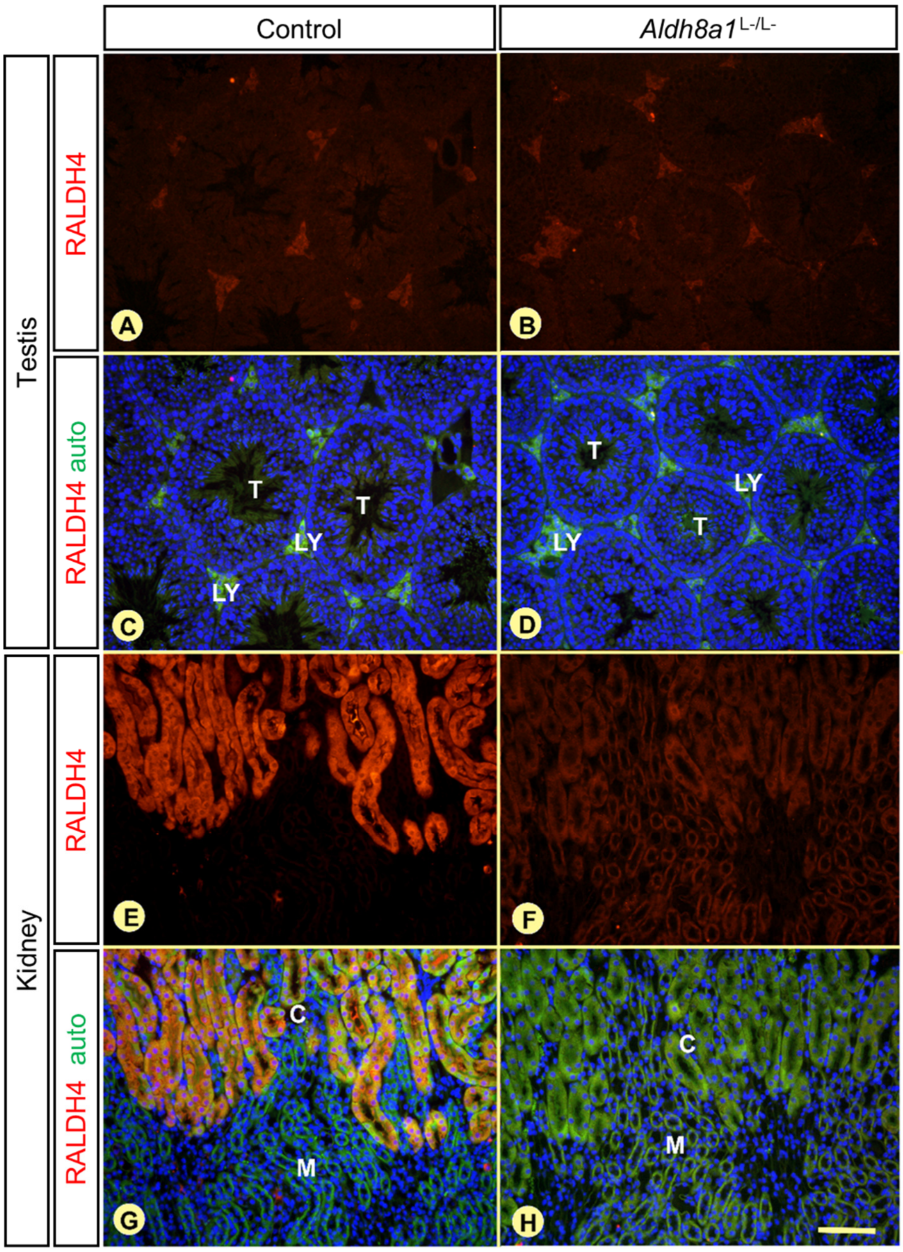

Fig. S9. Detection of RALDH4 in tissues from adult mouse males. (A-D) Histological sections though the testes of wild-type (A,C) and *Aldh8a1*$^{L-/L-}$ (B,D) mice, immunostained with antibodies to RALDH4. It is important to note that RALDH4 is absent from the seminiferous tubules (T) because this enzyme has the capacity to synthesize 9-*cis* retinoic acid, which binds to and activates RAR in vitro [(Lin et al, 2003), and references therein]. Note also that the faint red signal in Leydig cells (LY) corresponds to background staining as it is also present in the *Aldh8a1*$^{L-/L-}$ mutant testis. (E-H). Histological sections though the kidney of wild-type (E,F) and *Aldh8a1*$^{L-/L-}$ (G,H) mice, immunostained with antibodies to RALDH4. The wild-type kidney serves as a positive control of the procedure: RALDH4 is readily detected in the convoluted tubules localized in the kidney cortex (C), while absent from the kidney medulla (M), as previously reported (Lin et al, 2003). The absence of any signal in the *Aldh8a1*$^{L-/L-}$ mutant kidney demonstrates that the antibody is specific for RALDH4. (C,D,G and H) same sections as (A,B, E and F), respectively : the signal (red) was superimposed with the DAPI nuclear stain (blue) and with the tissue auto fluorescence (green). Scale Bar (H): 80 µm (A-H).

Development • Supplementary information

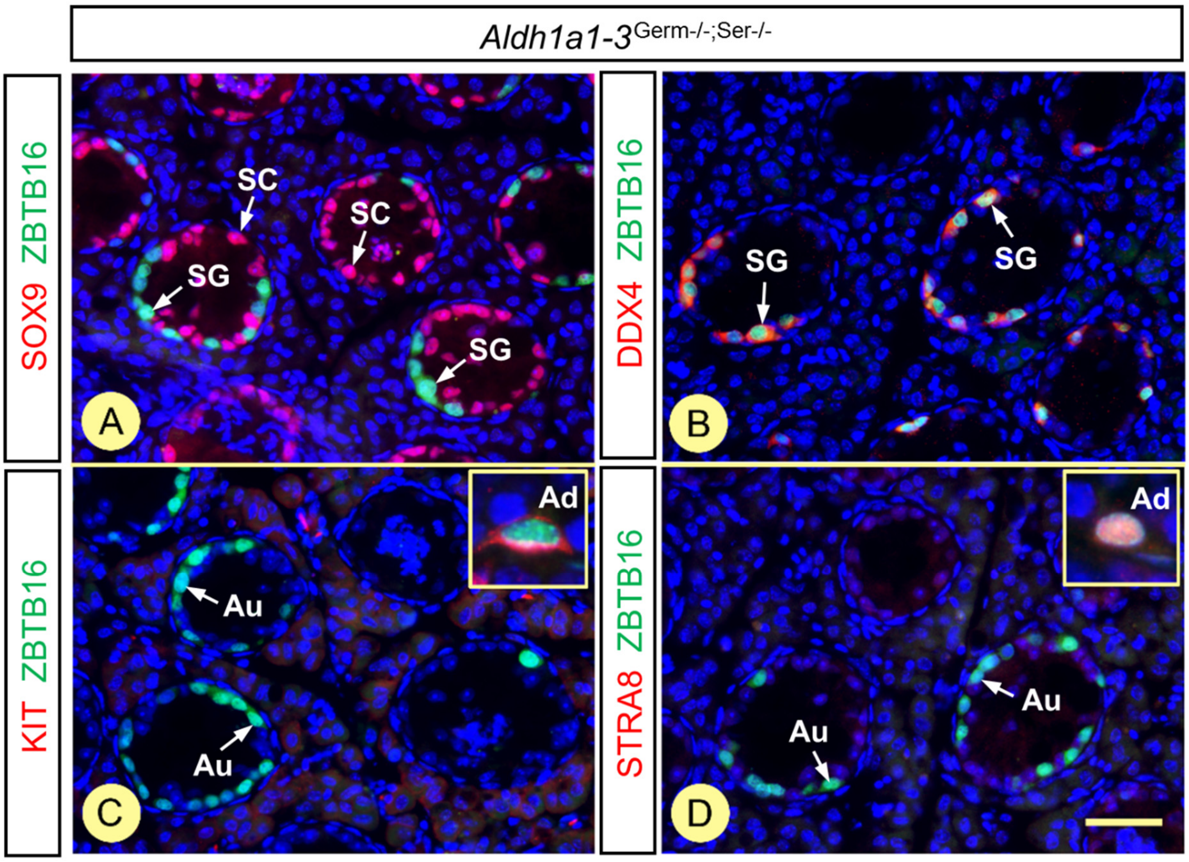

Fig. S10. Spermatogenesis is arrested in adult *Aldh1a1-3*$^{Germ-/-;\ Ser-/-}$ compound mutants. (A-D) Histological sections of 30 week-old testis immunostained with antibodies to: (A) SOX9, a marker of all Sertoli cells, (B) DDX4, a marker of all germ cell-types, (C) KIT and (D) STRA8, markers of differentiating spermatogonia, as indicated (red signals). In all cases, spermatogonia are also immunostained with antibodies to ZBTB16 (green signals). The insets (in C and D) correspond to examples of spermatogonia from a wild-type adult testis, used as a positive control of the immunostaining procedure. See Suppl. Table 1, IHC protocol 2 for technical details. Au, undifferentiated A spermatogonia; Ad, differentiating A spermatogonia; LY, Leydig cells; SC, Sertoli cells; SG, spermatogonia. Scale Bar in (D): 60 µm (A-D) and 9 µm (insets).

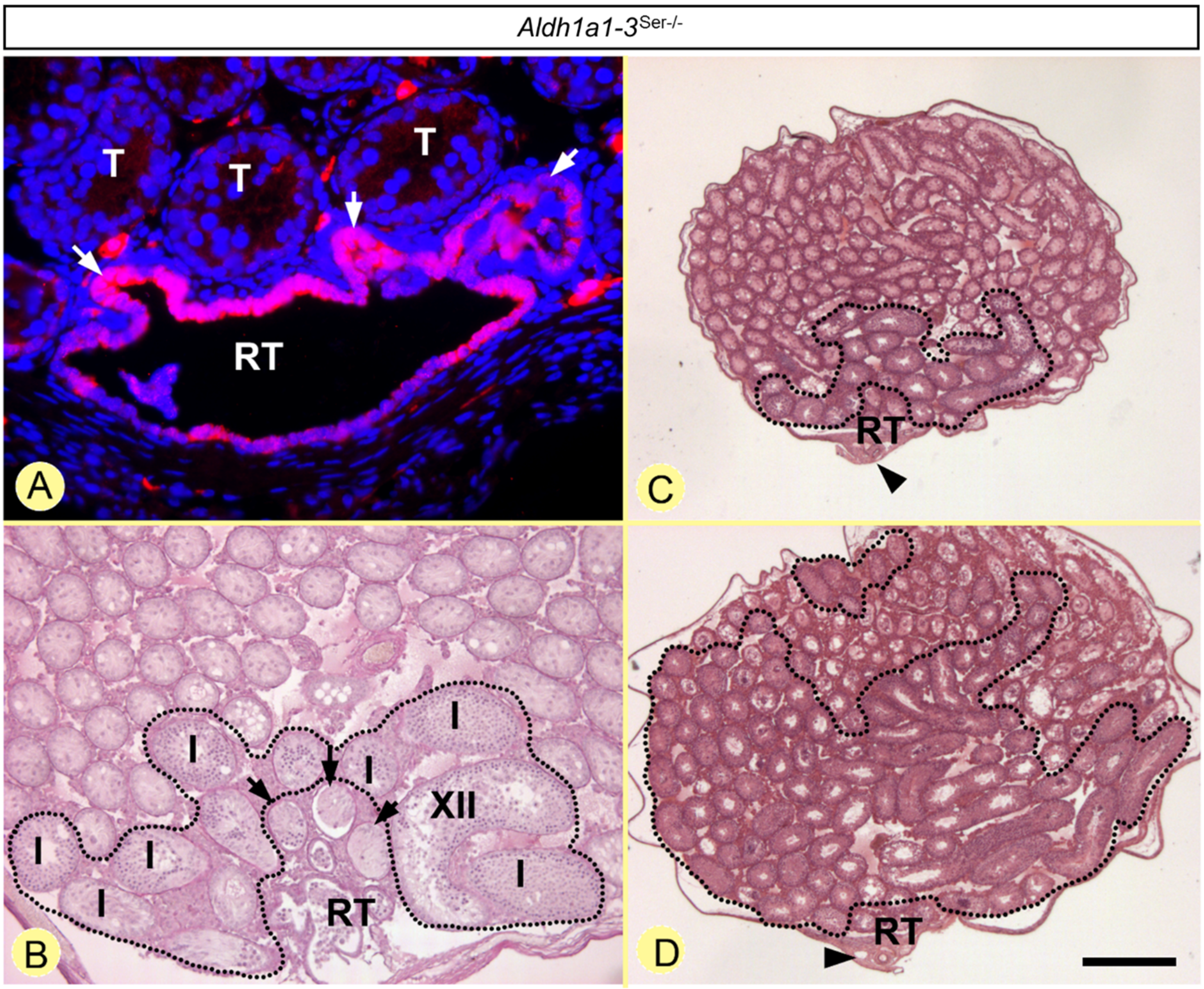

Fig. S11. Spontaneous recovery of spermatogenesis in ageing *Aldh1a1-3*$^{Ser-/-}$ mutants. (A) Detection of RALDH1 (red signal) in a 5 week-old *Aldh1a1-3*$^{Ser-/-}$ mutant: RALDH1 is present in epithelial cells of the rete testis (RT) and tubuli recti (arrows), but is absent from the epithelium of seminiferous tubules (T). Nuclei were counterstained with DAPI. (B) Transverse section through a 5 week-old mutant testis stained with PAS. The seminiferous tubule segments adjacent to the rete testis are synchronized either at stage I or at the stage which immediately precedes stage I in the SE cycle (i.e., stage XII). (C, D) Transverse sections through 5 (C) and 20 (D) week-old mutant testes stained with H&E: the seminiferous tubules cross-sections within the black dotted lines display spermatogenesis, whereas those localized outside contain only spermatogonia and Sertoli cells. The older is the mouse, the larger is the area displaying spermatogenesis. RT, rete testis. Arrows point to the tubuli recti and black arrowheads indicate the testis hilum. Scale bar (D): 50 µm (A), 160 µm (B) and 550 µm (C,D).

Unless exposed to exogenous ATRA, the SE of the *Aldh1a1-3*$^{Ser-/-}$ mutants contains only SC and A_{undiff} spermatogonia until the age of 4 weeks (Raverdeau et al., 2012). However, spontaneous recovery of spermatogenesis is observed in some *Aldh1a1-3*$^{Ser-/-}$ mutants aged 5 weeks and beyond (B). This recovery does not occur at random. Instead, segments of seminiferous tubules containing spermatocytes and/or spermatids at 5-6 weeks of age are always localized closest to rete testis, a cavity which collects all the seminiferous tubules (C). At 4-6 month of age, spermatogenesis has eventually recovered in a majority of the tubular segments, with the notable exception of those farthest from the rete (D). The most straightforward explanation for this phenomenon is the high level of RALDH1 expression (A) remaining in *Aldh1a1-3*$^{Ser-/-}$ mutant in epithelia lining the rete testis and tubuli recti, the short tubules connecting the seminiferous tubules proper to the rete testis.

Development • Supplementary information

Supplementary Materials and Methods

ISH with digoxigenin-labeled probes was as described (Vernet et al, 2006). The plasmids containing part of *Lgals1* (366 bp long; exons 2–4) and *Rec8* (766 bp long; exons 6-14) cDNAs were linearized and used as templates for the synthesis of riboprobes.

To construct the *Aldh8a1* targeting vector for homologous recombination in embryonic stem (ES) cells (see Suppl. Fig. 8), a 10 kb long fragment isolated from a 129/Sv mouse genomic DNA library and containing exons E5, was inserted into pBluescript II SK+ (Stratagene). A FRT-flanked neomycin resistance cassette (*neo*) was cloned downstream of exon 5. Oligonucleotides containing *lox*P sites, were then inserted on each side of exon 5. This exon (amino acid residues 198-283) encodes the aldehyde dehydrogenases glutamic acid active site (E residue at position 253 in the LELGGKNP motif) in RALDH4. A diphtheria toxin A (*DT-A*) expression cassette was inserted at the 3' side of the genomic DNA. This targeting vector was linearized and electroporated into ES cells. One clone, targeted as expected (out of 129), was identified by Southern blot analysis (K76, L3 allele, *Aldh8a1*$^{+/L3}$). Transient transfection of K76 ES cells with a FLP-expressing plasmid allowed excision of the *neo* gene, yielding cells bearing the conditional *lox*P-flanked L2 allele. One such clone (K76.101, L2 allele, *Aldh8a1*$^{+/L2}$) was injected into C57BL/6 blastocysts and the chimeras transmitted the conditional allele to their germ line. Homozygous mice (*Aldh8a1*$^{L2/L2}$) were indistinguishable from their wild-type littermates. They were crossed with *CMV-Cre* transgenic mice (Metzger and Chambon, 2001), and the resulting mice bearing the excised allele (*Aldh8a1*$^{+/L-}$) were identified by Southern blot and PCR analysis. Tail DNA was genotyped by PCR using primers 2 (5'-TTTATCACAGTGCAAAGCCCAGGGG-3') and 3 (5'-CACTCCAAGCTCAGAATGTGACAGG-3'; see Fig. 1A) to amplify the wild-type (341 bp-long) and the *lox*P-flanked L2 (411 bp-long), and using primers 1 (5'-GGTCACCTTGGTCTACAACTTCTGG-3') and 3 to amplify the excised, null, L– (400 bp long) allele (see Suppl. Fig. 8). Conditions were 30 cycles, with denaturation for 30 seconds at 94°C, annealing for 30 seconds at 61°C and elongation for 30 seconds at 72°C. Heterozygous mating (n=42; mean of seven pups per litter) yielded 25% (n=73) wild-type (*Aldh8a1*$^{+/+}$), 50% (n=146) heterozygous (*Aldh8a1*$^{+/L-}$) and 25% (n=75) homozygous (*Aldh8a1*$^{L-/L-}$) mice. Both mutant males and females grew normally, were fertile, healthy and indistinguishable from wild-type littermates. Altogether, these results indicate that RALDH4 is not indispensable for post-natal life and fertility.

Supplemental references

Ahmed, E.A., de Rooij, D.G. (2009). Staging of Mouse Seminiferous Tubule Cross-Sections. In: Keeney S. (eds) Meiosis. Methods in Molecular Biology (Methods and Protocols), vol 558. Humana Press, Totowa, NJ.

Bouillet, P., Sapin, V., Chazaud, C., Messaddeq, N., Décimo, D., Dollé, P. and Chambon. P. (1997). Developmental expression pattern of Stra6, a retinoic acid-responsive gene encoding a new type of membrane protein. *Mech Dev.* **63**, 173-186.

Hamer, G., Roepers-Gajadien, H. L., van Duyn-Goedhart, A., Gademan, I. S., Kal, H. B., van Buul, P. P. and de Rooij, D. G. (2003). DNA double-strand breaks and gamma-H2AX signaling in the testis. *Biol. Reprod.* **68**, 628-634.

Lin, M., Zhang, M., Abraham, M., Smith, S.M., Napoli, J.L. (2003). Mouse retinal dehydrogenase 4 (RALDH4), molecular cloning, cellular expression, and activity in 9-cis-retinoic acid biosynthesis in intact cells. *J. Biol. Chem.* **278**, 9856-9861.

Mark, M., Teletin, M., Vernet, N. and Ghyselinck, N. B. (2015). Role of retinoic acid receptor (RAR) signaling in post-natal male germ cell differentiation. *Biochim Biophys Acta* **1849**, 84-93.

Matt, N., Dupe, V., Garnier, J. M., Dennefeld, C., Chambon, P., Mark, M. and Ghyselinck, N. B. (2005). Retinoic acid-dependent eye morphogenesis is orchestrated by neural crest cells. *Development* **132**, 4789-4800.

Metzger, D. and Chambon, P. (2001). Site- and time-specific gene targeting in the mouse. *Methods* **24**, 71-80.

Oulad-Abdelghani, M., Bouillet, P., Décimo, D., Gansmuller, A., Heyberger, S., Dollé, P., Bronner, S., Lutz, Y., Chambon, P. (1996). Characterization of a premeiotic germ cell-specific cytoplasmic protein encoded by Stra8, a novel retinoic acid-responsive gene. *J. Cell Biol.* **135**, 469-477.

Raverdeau, M., Gely-Pernot, A., Féret, B., Dennefeld, C., Benoit, G., Davidson, I., Chambon, P., Mark, M., and Ghyselinck, N.B. (2012). Retinoic acid induces Sertoli cell paracrine signals for spermatogonia differentiation but cell autonomously drives spermatocyte meiosis. *Proc. Natl. Acad. Sci. USA,* **109**, 16582-16587.

Vernet, N., Dennefeld, C., Guillou, F., Chambon, P., Ghyselinck, N. B. and Mark, M. (2006). Prepubertal testis development relies on retinoic acid but not rexinoid receptors in Sertoli cells. *EMBO J.* **25**, 5816-5825.
